# Differences in technical and clinical perspectives on AI validation in cancer imaging: mind the gap!

**DOI:** 10.1186/s41747-024-00543-0

**Published:** 2025-01-15

**Authors:** Ioanna Chouvarda, Sara Colantonio, Ana S. C. Verde, Ana Jimenez-Pastor, Leonor Cerdá-Alberich, Yannick Metz, Lithin Zacharias, Shereen Nabhani-Gebara, Maciej Bobowicz, Gianna Tsakou, Karim Lekadir, Manolis Tsiknakis, Luis Martí-Bonmati, Nikolaos Papanikolaou

**Affiliations:** 1 School of Medicine, Aristotle University of Thessaloniki, Thessaloniki, Greece; 2 Institute of Information Science and Technologies of the National Research Council of Italy, Pisa, Italy; 3 Computational Clinical Imaging Group (CCIG), Champalimaud Research, Champalimaud Foundation, Lisbon, Portugal; 4 Quibim SL, Valencia, Spain; 5 Biomedical Imaging Research Group (GIBI230), La Fe Health Research Institute, Valencia, Spain; 6 Data Analysis and Visualization, University of Konstanz, Konstanz, Germany; 7 Department of Pharmacy, Kingston University London, London, UK; 8 Faculty of Health, Science, Social Care & Education, Kingston University London, London, UK; 9 2nd Department of Radiology, Medical University of Gdansk, Gdansk, Poland; 10 Research and Development Lab, Gruppo Maggioli Greek Branch, Maroussi, Greece; 11 Departament de Matemàtiques i Informàtica, Artificial Intelligence in Medicine Lab (BCN-AIM), Universitat de Barcelona, Barcelona, Spain; 12 Institució Catalana de Recerca i Estudis Avançats (ICREA), Barcelona, Spain; 13 Computational BioMedicine Laboratory (CBML), Foundation for Research and Technology-Hellas (FORTH), Heraklion, Greece; 14 Radiology Department, La Fe Polytechnic and University Hospital and Health Research Institute, Valencia, Spain

**Keywords:** Artificial intelligence, Diagnostic imaging, Neoplasms, Research design, Surveys and questionnaires

## Abstract

**Abstract:**

Good practices in artificial intelligence (AI) model validation are key for achieving trustworthy AI. Within the cancer imaging domain, attracting the attention of clinical and technical AI enthusiasts, this work discusses current gaps in AI validation strategies, examining existing practices that are common or variable across technical groups (TGs) and clinical groups (CGs). The work is based on a set of structured questions encompassing several AI validation topics, addressed to professionals working in AI for medical imaging. A total of 49 responses were obtained and analysed to identify trends and patterns. While TGs valued transparency and traceability the most, CGs pointed out the importance of explainability. Among the topics where TGs may benefit from further exposure are stability and robustness checks, and mitigation of fairness issues. On the other hand, CGs seemed more reluctant towards synthetic data for validation and would benefit from exposure to cross-validation techniques, or segmentation metrics. Topics emerging from the open questions were utility, capability, adoption and trustworthiness. These findings on current trends in AI validation strategies may guide the creation of guidelines necessary for training the next generation of professionals working with AI in healthcare and contribute to bridging any technical-clinical gap in AI validation.

**Relevance statement:**

This study recognised current gaps in understanding and applying AI validation strategies in cancer imaging and helped promote trust and adoption for interdisciplinary teams of technical and clinical researchers.

**Key Points:**

- Clinical and technical researchers emphasise interpretability, external validation with diverse data, and bias awareness in AI validation for cancer imaging.
- In cancer imaging AI research, clinical researchers prioritise explainability, while technical researchers focus on transparency and traceability, and see potential in synthetic datasets.
- Researchers advocate for greater homogenisation of AI validation practices in cancer imaging.

**Graphical Abstract:**

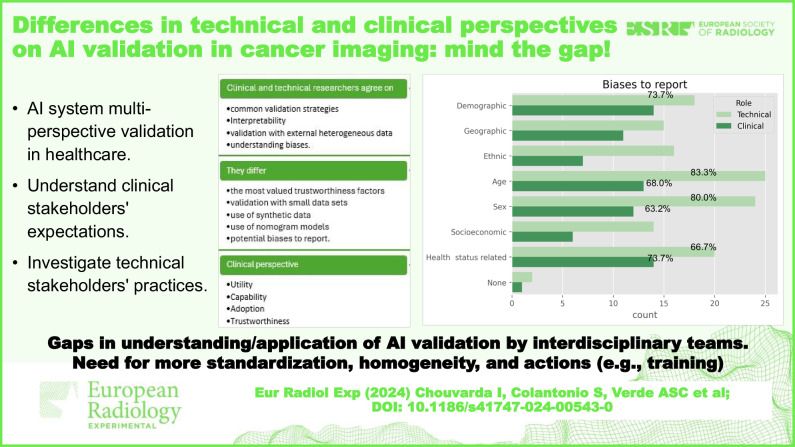

## Background

Artificial intelligence (AI) is increasingly being used in medical imaging, particularly in cancer imaging. This involves integrating and analysing big and complex data, including medical images and clinicobiological information, to support critical decision-making in various oncology tasks, including risk assessment, screening, diagnosis, organ segmentation, radiology-pathology correlation, treatment response prediction, and multi-omics data integration [1]. These scientific endeavours of AI development are typically interdisciplinary and require rigorous and extensive validation.

Limited research exists on practitioners’ attitudes and experiences with AI tools. As outlined by Jungmann et al [2], despite the common belief in the potential positive impact of AI on healthcare, there is often low confidence (25%) in its reliability. There is disagreement on medical societies’ involvement in clinical validation, informing patients about the use of AI, and its inclusion in medical education.

Trustworthiness and clinical validation, with quality reporting and explanation, are crucial for the acceptance of AI systems. In 2018, a call for the robust clinical validation of new AI tools was published [3]. Yin et al [4] compiled a systematic review of 51 studies of AI applications evaluated in healthcare (screening, diagnosis, risk analysis, and treatment), and found that 26 studies looked at AI performance, 33 at clinician outcomes, 14 at patient outcomes and one at the economic impact.

There have been multiple efforts in the research community toward standardised AI development and validation in healthcare/medical imaging and to promote the standardisation of AI-based research reporting. The CLAIM [5] has proposed a checklist for reporting AI applications in medical imaging, and the CLEAR checklist for radiomics research [6]. The R-AI-DIOLOGY checklist [7] assesses the trustworthiness of technical solutions in clinical neuroradiology, covering ten aspects such as disease definition, case selection, data acquisition, quality checks, anonymisation, data management, integration, tool updates, validation, labels, and ground truth datasets for radiologist workflow.

Regarding AI trustworthiness, a framework was proposed by the European Commission’s High-Level Expert Group on AI [8], focusing on seven key requirements for trustworthiness (AI technical robustness and safety, human agency and oversight, privacy and data governance, transparency, diversity, non-discrimination and fairness, societal/environmental well-being, and accountability), along with a related assessment list (ALTAI) [9]. However, the framework is generic and requires further specification for domains such as cancer imaging.

A more recent initiative related to the development and validation of trustworthy AI in the medical field is the FUTURE-AI framework [10], based on a broad consensus (over 100 collaborators worldwide), which proposes 30 actionable guidelines for trustworthy AI in the medical field [11]. These guidelines, based on six guiding principles for trustworthy AI (fairness, universality, traceability, usability, robustness, and explainability), cover the entire AI lifecycle, from design to deployment and monitoring, and consider technical, clinical, legal, and socioethical aspects of AI.

Finally, a recent work [12] presents an evaluation framework called “Translational Evaluation of Healthcare AI” (TEHAI) to guide the implementation of AI systems in healthcare settings. After identifying gaps in existing evaluation and reporting frameworks, the final framework proposes three main components: capability, utility, and adoption. Wider adoption of these standards could facilitate coordination between technical and clinical research.

Despite these valuable efforts, gaps still exist. A study following the CLAIM publication [13], reported deviations from the CLAIM guidelines in a large proportion of AI studies, for example, regarding lack of information on data management, ground truth reference, inter- and intra-reader variability, interpretability, and model failure analysis, shortcomings also identified in the review of Alabed et al [14]. A recent study [15] found a lack of consistency and robustness among well-known guidelines in AI for medical imaging, as well as difficulty in compliance due to their length and complexity, as well as the field’s fast-evolving nature.

This paper examines gaps in AI validation and reporting in health/cancer imaging, identifying common practices, strategy variability, and potential causes. It identifies technical, clinical, and other barriers preventing multiperspective validation and divergence between technical and clinical experts in expectations and information in AI validation reports. This work is performed within the AI for health imaging (AI4HI) network, which consists of partners involved in five large EU-funded projects on big data and AI in cancer imaging: CHAIMELEON [16], EUCANIMAGE [17], INCISIVE [18], ProCancer-I [19], and PRIMAGE [20]. This network collectively uses big data repositories of annotated cancer images from hundreds of thousands of cancer patients to develop AI solutions for cancer diagnosis, treatment, and follow-up. It includes a wide range of stakeholders, perspectives, approaches, and disciplines, including technical, clinical, legal, ethical, and more. The network’s AI and clinical expertise provide insights into their attitudes and strategies towards AI validation.

This analysis aims to understand clinical stakeholders’ expectations and investigate technical stakeholders’ practices regarding AI system multi-perspective validation in healthcare. This can set the basis and fuel further discussion on actions towards bridging the gap in AI validation practices between technical and clinical experts, with the mid-term goal of contributing to practical guidelines for harmonisation of validation methodologies and reporting guidelines/tools for AI in cancer imaging.

## Methods

In the context of the AI Validation Working Group of the AI4HI network, a set of structured questions was proposed and organised into sections to address several AI validation topics. Technical and clinical experts of the network were asked to provide their responses. This wealth of expertise in the AI4HI network allowed us to collect responses from 49 individuals involved in AI development and validation from technical and clinical perspectives.

### Questionnaire design and description

The AI-validation set of questions was designed collaboratively by defining the different validation themes to be covered and the relevant questions that could help identify the attitudes and practices of technical and clinical users. Most questions allowed for one or more potential answers, defined and improved in several iterations during a series of joint biweekly meetings. A glossary of terms was included in the questionnaire to facilitate the proper understanding of the questions by different professional profiles and avoid potential ambiguities, *e.g.*, between explainability and interpretability [21], as distinct terms [22] (Supplemental File 1). A small set of open-ended questions was added, allowing for free-form answers to enable capturing the knowledge, expertise, and understanding of the respondents. The option “out of my expertise”, and ‘other’ were included as a possible reply to several questions.

The consolidated structure consists of six sections. A detailed presentation is available in Supplemental File 2, while the sections are briefly described below

1. The ‘Profile’ section contains questions about the respondent’s profile (age, expertise, work placement, and location) and the method(s) of involvement in the AI research (clinical expert, data provider and curation, model training, and technical/clinical model validation).
2. In the ‘Overall strategy for model building and validation’ section, questions relate to practices during AI model development that impact model validation and address both technical and clinical researchers. These aspects include model trustworthiness, robustness, generalizability, the potential trade-off between model interpretability and performance, and validation strategies at an early stage to promote its applicability in a realistic clinical setting.
3. In the ‘Technical validation’ section (optional for clinicians), respondents are asked to reflect on the technical approaches used in the creation and evaluation of AI models using internal and external datasets [23]. The questions refer to approaches of varying complexity.
4. The ‘Statistical analysis and evaluation metrics’ section addresses the user’s practices related to the metrics used for statistical analysis of binary classification, segmentation and regression models, taking into account the overall objective of each model.
5. The ‘Bias and fairness’ section addresses the user’s perspective on detecting, reporting, and adjusting biases in the AI model (*e.g.*, due to under-represented groups in the training data) and their relationship to the fairness of the model.
6. Finally, in the ‘Concluding questions’ section, the user can indicate their approach to AI validation and suggest important topics in this context.

### Data collection

The set of structured questions was implemented as an electronic questionnaire distributed via a web link to partners involved in AI technical or clinical validation in the five AI4HI projects. Participation was anonymous and voluntary and all AI4HI partners were invited to participate in these joint activities, respecting privacy and confidentiality standards. No internet protocol-IP address was recorded together with the answers.

In this phase, the aim was not to reach out to the AI community at large but rather to have a limited set of answers from respondents that were filtered based on their involvement in the domain, avoiding the noise of unattended responses.

### Data analysis

Most of the questions had a closed form and were therefore approached with a quantitative analysis. For each question, the frequency of individual responses and grouped responses was analysed and compared between user groups. Respondents were categorised into user groups based on their role(s) in the research project(s), using their combined responses, in case of multiple roles within the project. Each combination of roles results in a single possible user group, which can be either clinical or technical (see Table C1). An exploratory analysis was performed using R v4.2.2 software and Python 3.9.0. We performed descriptive statistics for all respondents and separately per user group by first identifying the most common characteristics of the user profile. We then created simple or stacked bar charts for single or multiple-choice questions and determined the percentage of responses within each category. A Pearson χ^2^ test was also performed between TG and CG, to highlight statistically significant differences in responses.

The responses to the open question (Other than the AI-validation-related topics above, are there other AI validation perspectives, especially from a clinical point of view, that you think are important for an AI validation plan to cover?) were imported into data management software called NVivo 12. The six phases of thematic analysis based on Braun and Clarke [24] were used to analyse the qualitative data, keeping in mind the TEHAI components to structure our qualitative analysis.

## Results

In total, responses to the AI-validation questions were collected from 49 AI4HI participants over 2 months, which corresponded to a 70% response rate, approximately. The majority (59.2%) were in the range of 20–40 years of age, followed by the range of 40–60 (34.7%). Their work experience was more frequently 1–5 years (40.8%), while groups of 5–10 years and 10–20 years were also well represented (28.6% and 26.5%, respectively). Most of the respondents work in public research institutes (69.4%), but health organisations (22.4%) and companies (8.2%) were also represented. Most responses came from the combination ‘young researchers in public institutes’, but other combinations were also represented (see Table C2 in Supplemental File 3). The age above 60 was less represented, and the combination “above 60 and private company” was absent.

A total of 30 respondents constituted the technical group (TG), while 19 respondents were allocated to the clinical group (CG). Figure C1 in Supplemental File 3 describes the frequency of roles within the AI project, where multiple responses were possible. Each unique combination of roles categorises the respondent into the TG or CG.

The following subsections present the main differences and agreements in responses between the two groups, in-depth findings about each group, and other themes that emerged.

### Comparison between the TG and the CG

Overall, the 15 questions that presented statistically significant differences (χ^2^ *p*-value < 0.05) between TG and CG are summarised in Table 1. They revolve around the topics: balance interpretability and performance, ground truth, factors for trustworthy models, the strategy for explainability, improving generalizability, pre-checks on new data, model error analysis, reasons behind wrongly classified data, use of small datasets, how to evaluate models, validation with external datasets, metrics for segmentation models, metrics for regression models, potential biases, strategies to validate models. These differences are discussed in detail below.

**Table 1:** The questions that present statistically significant differences between CG and TG

Question number	Topic	*p*-value
7	Balance interpretability and performance	0.001
9	Ground truth	0.007
10	Factors for trustworthy models^*^	0.007
11	Strategy for explainability^*^	0.008
12	Improving generalizability	0.002
13	Pre-checks on new data/limitations	0.002
14	Model error analysis	< 0.001
15	Reasons behind wrongly classified data	0.001
19	Small dataset-nested cross-validation	< 0.001
20	Evaluate models^*^	< 0.001
22	Validation with external datasets	0.005
25	Metrics to validate segmentation models^*^	< 0.001
26	Metrics for regression models^*^	< 0.001
33	Potential biases^*^	0.0395
34	Strategies to validate your models^*^	< 0.001

^*^ Denotes a question with multiple possible answers

**Fig. 1:**
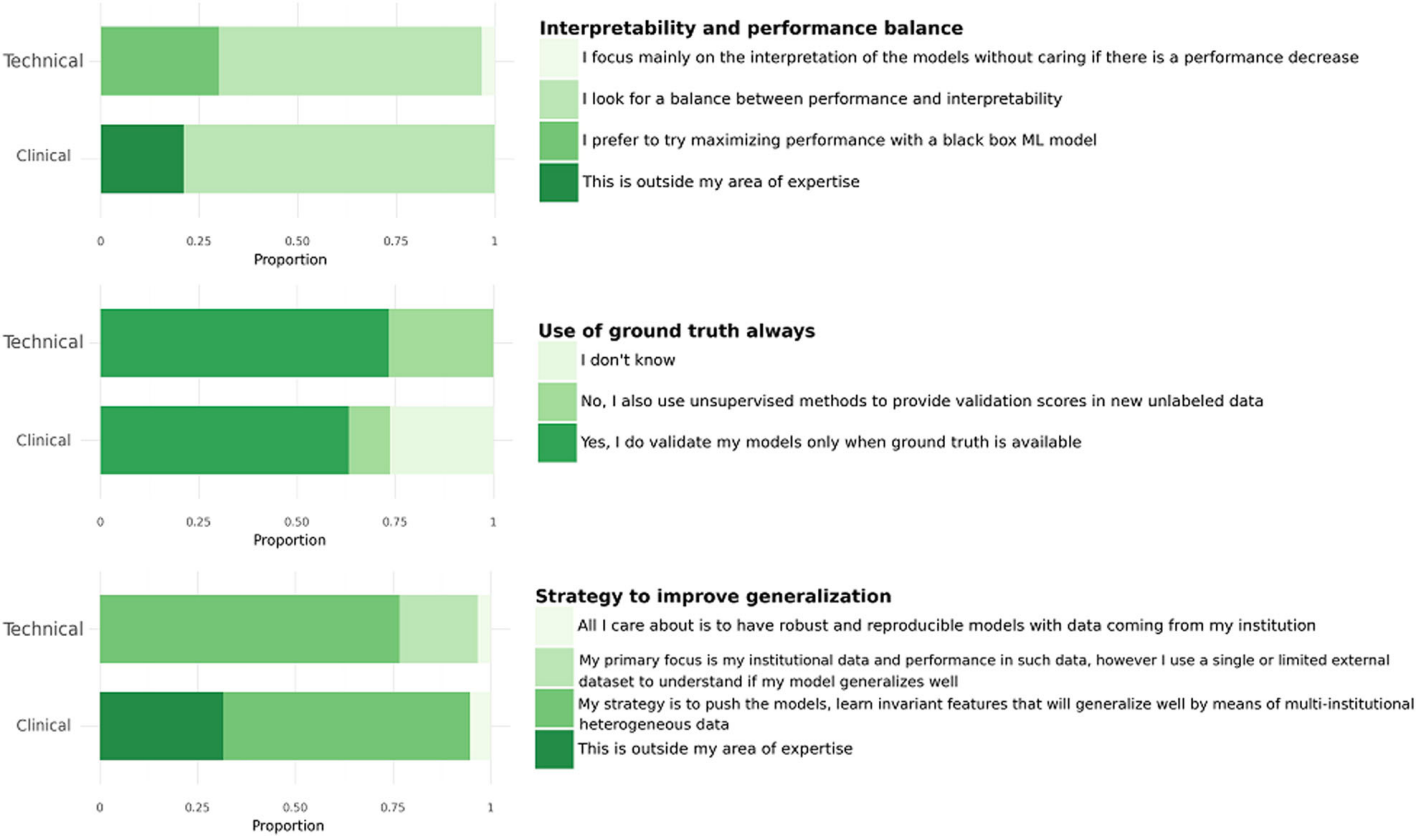
‘Overall strategy for model building and validation’ topics comparing the points of view of the TG and the CG. (Top) Balance between Interpretability and Performance. (Middle) Always use ground truth. (Bottom) Strategy to improve generalisation

In relation to the ‘Overall strategy for model building and validation’ (Fig. 1) in both TG and CG, the majority of the participants (66.7% in TG and 78.9% in CG) looked for a balance between interpretability and performance in model building, which can be considered as expected in the medical domain. It was also observed that both groups agreed on validating their models only when ground truth was available (77.3% in TG and 63.2% in CG). A minority of participants also considered the use of unsupervised methods to provide validation scores in new unlabelled data (26.7% in TG and 10.5% in CG). In relation to the strategy to improve generalizability, there was agreement on prioritising the use of multi-institutional heterogeneous data (76.7% in TG and 63.2% in CG), which confirms the choices made in AI4HI research projects and other relevant initiatives [25].

**Fig. 2:**
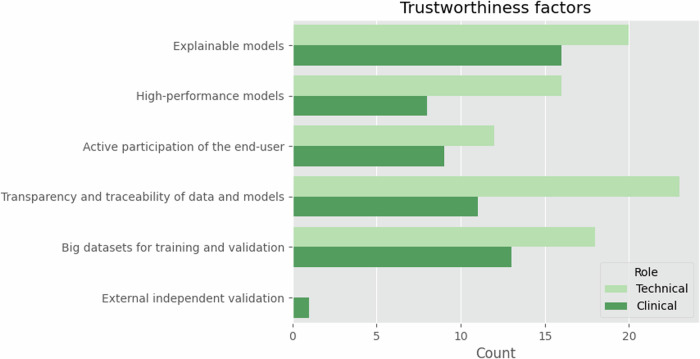
The factors to consider a model as trustworthiness, comparing the points of view of the TG and the CG

Regarding the factors to consider models trustworthy (Fig. 2), it is observed that in TG, 76.7% of the technical participants gave more relevance to the transparency and traceability of data and models, *i.e.*, a technical aspect, this factor being the third most important for CG (57.9%). In contrast, explainable models [26] were of higher interest for CG, with 84.2% of the votes being the second most voted choice in TG (66.7%).

Regarding ‘Technical validation’ (Fig. C2 in Supplemental File 3), a variety of opinions were recorded. When dealing with small datasets (< 200 patients), while both groups considered 80% of the data and the remaining 20% for testing, the preferred validation strategy by TG (53.33%) was to use k-fold cross-validation, while in CG, the most voted option (42.1%) was a single split. With respect to the use of new datasets for external validation, both groups reported usually using such new data when possible (86.7% in TG and 52.6% in CG). However, in the CG, 36.8% of the participants never performed external validation. Finally, 80.0% of TG and 68.4% of CG participants used only real data during validation, with only 20.0% in TG and 21.0% in CG using both real and simulated/synthetic data after assessing the validity of the synthetic data.

Concerning the specific methods used for model validation, all TG agreed on the use of performance metrics, and most of CG (52.6%) voted the same. Both groups considered the use of visual analytics tools as the second most-used option (46.7% in the TG and 31.6% in the CG), with manual checks in the last position (36.7% in the TG and 21.0% in the CG).

**Fig. 3:**
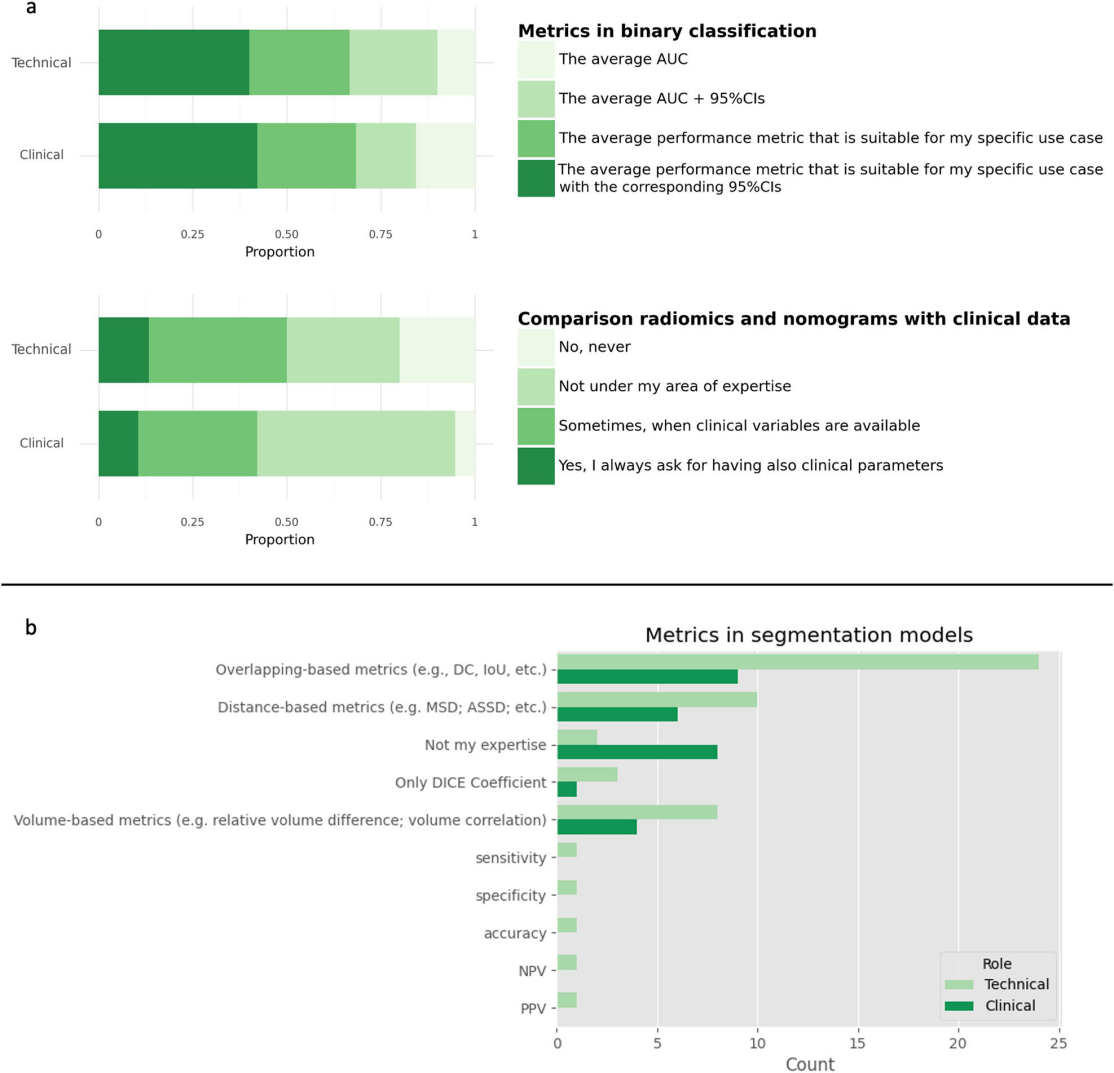
Stacked bar plots (**a**) and multiple-choice counts (**b**) on some ‘Statistical analysis and evaluation metrics’ topics comparing the points of view of the TG and the CG

In relation to the ‘Statistical analysis and evaluation metrics’ (Fig. 3), when evaluating a classification model, both groups preferred the average performance metric suitable for the specific use case with the corresponding 95% confidence interval (40.0% for TG and 42.1% for CG), which reflects the level of compliance with previously suggested reporting guidelines [5]. For segmentation models, using the DICE coefficient and other metrics that measure the overlap between the predicted mask and the ground truth was the most voted option in both groups (80.0% in TG and 47.4% in CG). In regression models, the mean square error (MSE) was the most voted option in both TG (66.7%) and CG (42.1%). The study also found that 42.1% of CG and 50.0% of TG would compare the usefulness of a radiomics model against nomograms built only on clinical variables, while 52.6% of CG and 30.0% of TG claimed a lack of expertise in such comparisons.

**Fig. 4:**
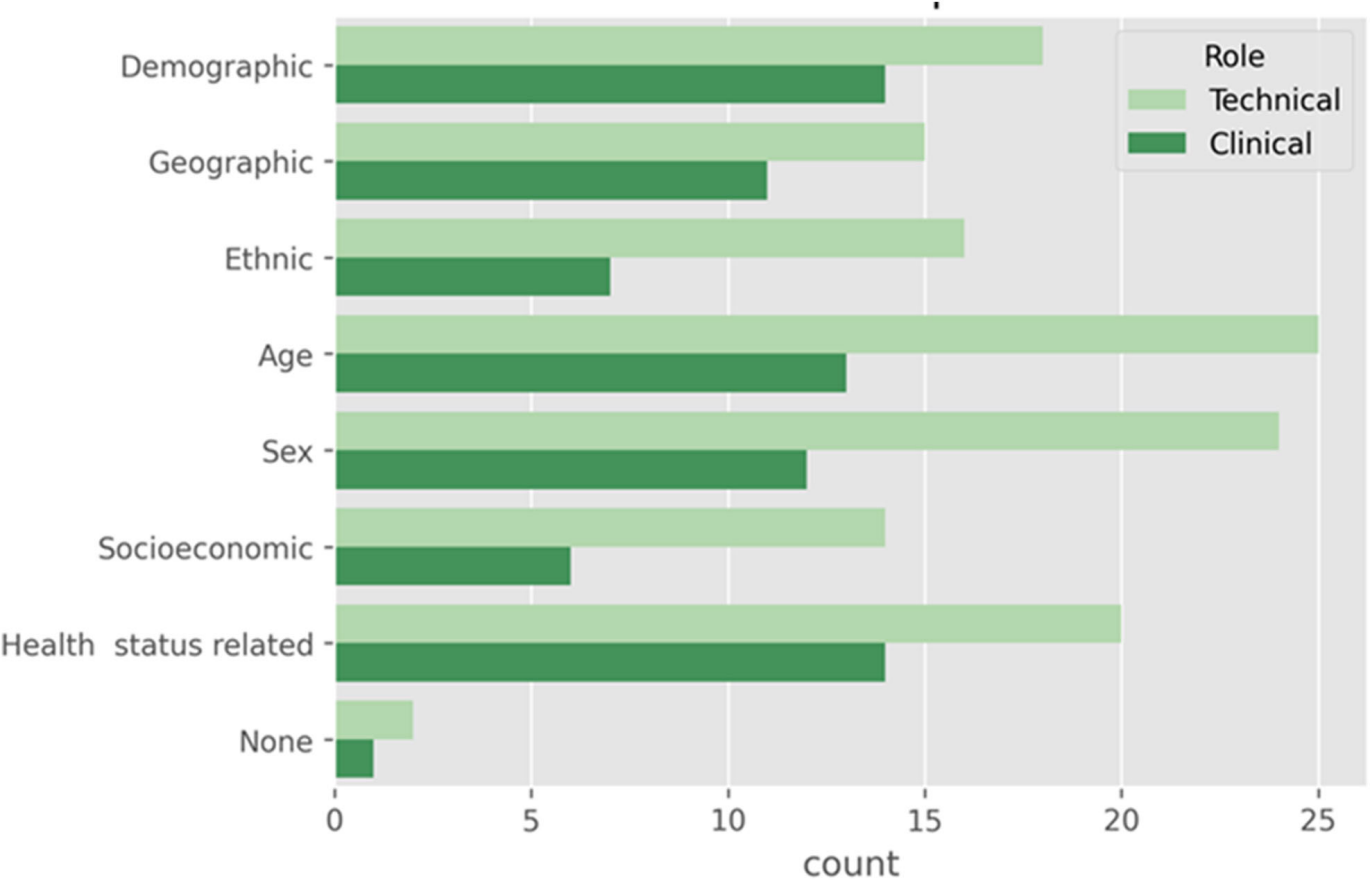
The potential biases to report, comparing the points of view of the TG and the CG

Regarding the ‘Bias and fairness’ (Fig. C3 in Supplemental File 3) of the models, both groups in general demonstrated awareness of selection bias in the collected datasets, with a small minority in TG stating a lack of knowledge. A total of 53.3% of the TG and 52.6% of CG participants used methods to mitigate this bias. Concerning the presence of biases in the dataset, 93.3% and 68.4% of the TG and CG participants, respectively, evaluated and reported it. When evaluating a trained model, both groups evaluated the fairness of the models for demographic, geographic, ethnicity, age, sex, and socioeconomic biases (66.7% in TG and 73.7% in CG). When listing the potential biases that need to be reported (Fig. 4), we found a balanced distribution among all the options, with age being the most voted option in TG (83.3%), followed by sex (80.0%) and health status-related information (66.7%). In the CG, the most voted biases were demographic and health status-related information (73.7% each), followed by age (68.0%) and sex (63.2%).

**Fig. 5:**
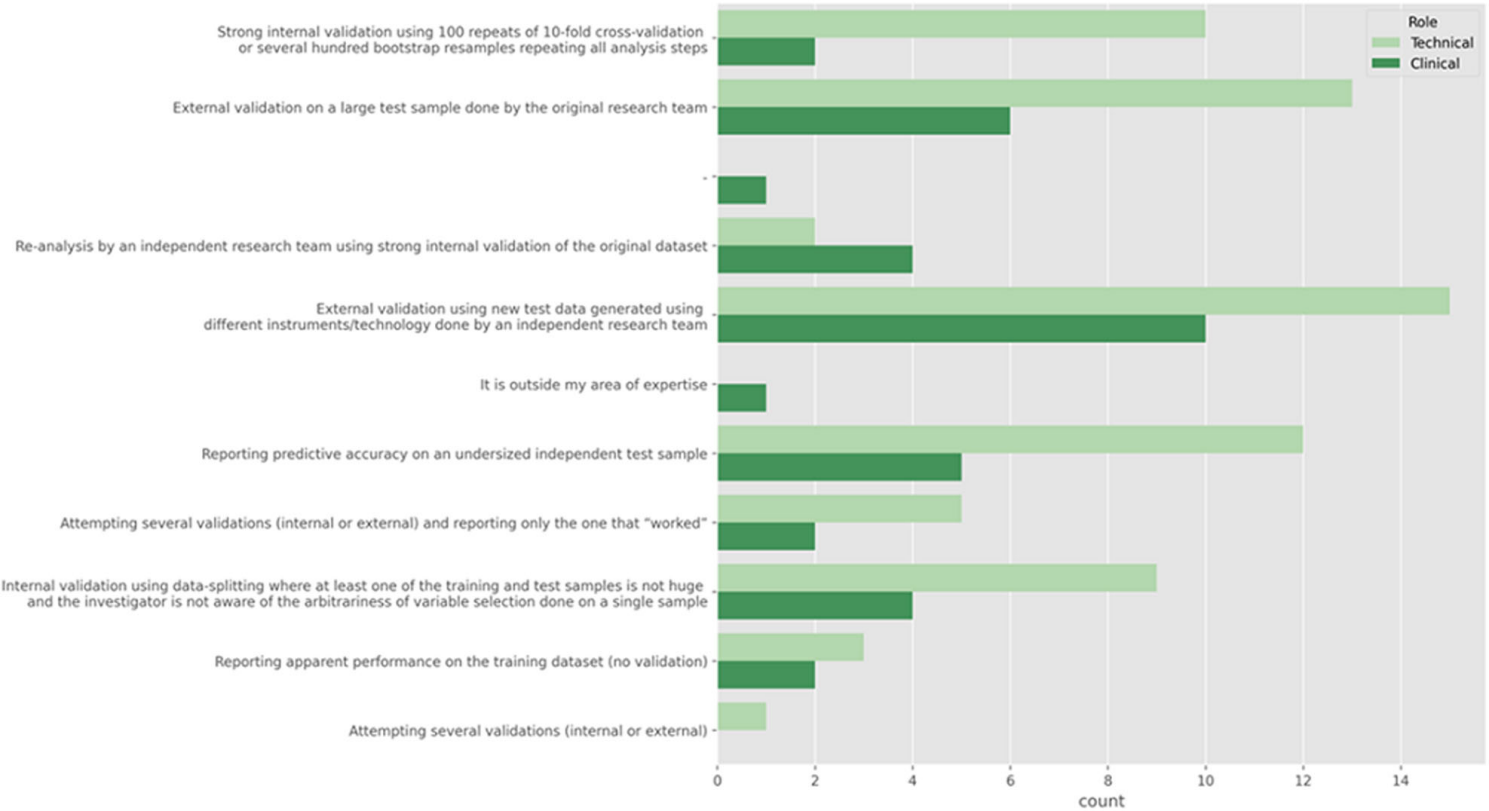
The main strategies followed by the participants when validating an AI model, comparing the TG and the CG

Finally, with regards to the general strategy followed when validating an AI model (Fig. 5), the TG preferred the external validation on a large test sample (46.7%), followed by reporting predictive accuracy on an undersized independent test sample (36.7%), while in CG, these options were selected in 31.6% and 26.3% of the cases, respectively, and equally with 31.6%, was the external validation by an independent research team. Therefore, both groups agree on the most common validation strategies.

### In-depth findings for the TG

This section focuses on an additional in-depth analysis of responses received from TG (30 respondents, most of them young researchers, 20–40-years-old and 1–5 years of professional expertise, employed in European public research institutions) (see Fig. D1 in Supplemental File 4).

**Fig. 6:**
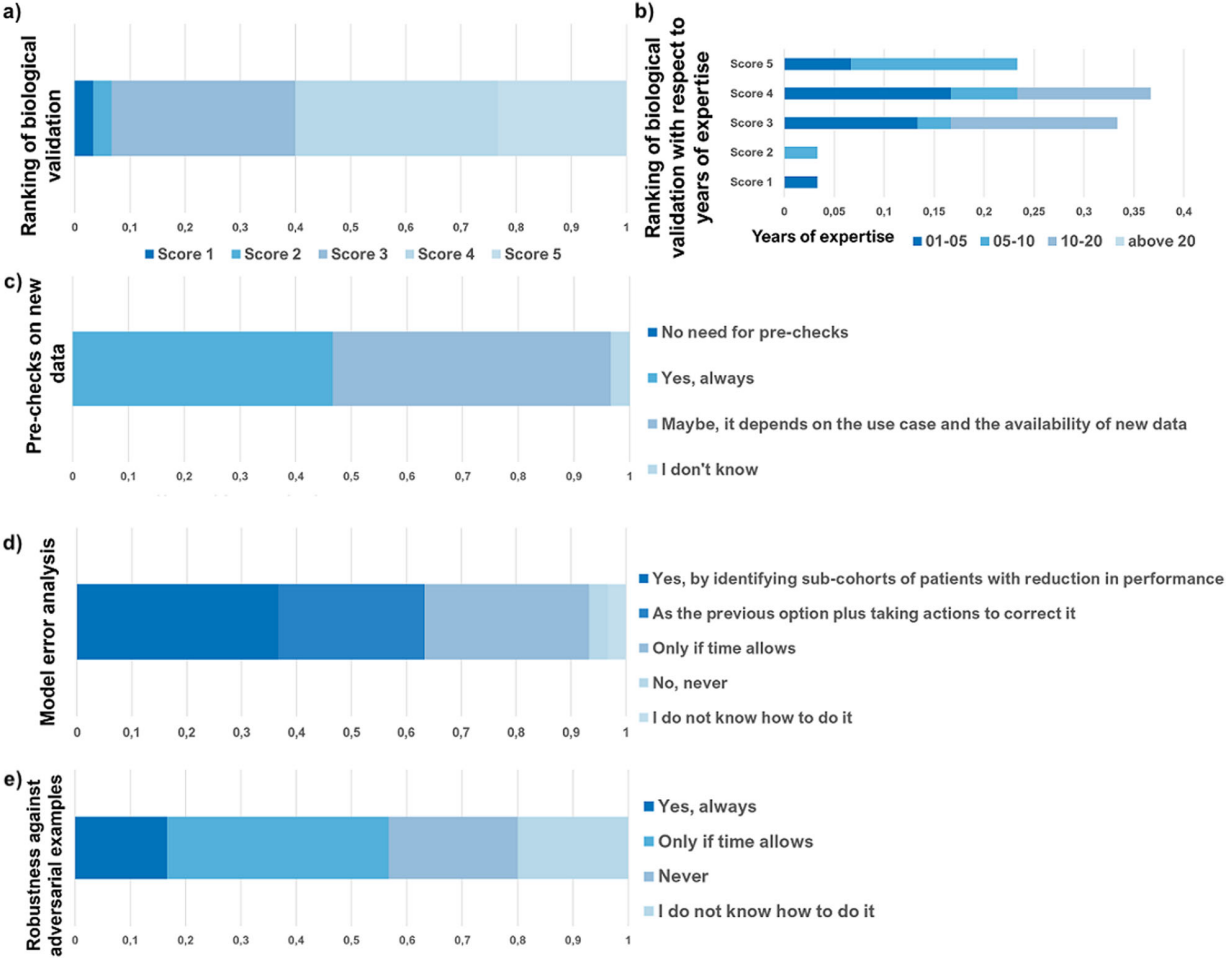
‘Technical validation’ topics for the TG respondents. All the plots are in 0-to-1 proportions. **a** Ranking of biological validation; **b** Ranking of bioloigcal validation with respect to years of expertise; **c** Pre-checks on new data; **d** Model error analysis; **e** Robustness against adversarial examples

In relation to the ‘Overall strategy for model building and validation’, the vast majority of TG considered biological validation to be highly important (see Fig. 6a), with the larger share scoring its importance as 4 (36.7%). It is worth noting that respondents with higher levels of expertise (*i.e.*, between 10 years and 20 years) only gave an average or good score in relation to the importance of biological validation (*i.e.*, 3 and 4), while the top score originated mainly from respondents with less than 10 years of expertise (see Fig. 6b), which might be related to increasing awareness of this issue among the younger AI developers.

Regarding explainability, the use of *post hoc* methods based on important features was the most common approach (73.3%), while the provision of local explanations was slightly less common (60.0%). Interestingly, the use of both approaches was quite commonly considered (46.7%) and only a small proportion of respondents (16.7%) indicated a lack of expertise. Pre-checks of new data were given due consideration by TG respondents, although only 50% stated that they always perform them (see Fig. 6c). Favourably, most respondents (63.3%) confirmed always performing some form of error analysis, with a proportion of them (26.7%) also taking corrective action (see Fig. 6d). However, a significant proportion of respondents (33.3%) indicated that they only perform error analysis when time permits or when the performance of the model is unsatisfactory (an option added by one respondent). Similarly, all TG respondents seemed to usually analyse the reasons for their AI model’s misbehaviour, although most of them (53.3%) stated that they would only perform such analyses if time allowed. This may require further attention, as identifying conditions that affect model performance is important to ensure model trustworthiness. Robustness to adversarial examples appeared to receive little attention from TG respondents (always checked by 16.7%), while a significant proportion stated that they only looked at this issue when time allowed or never (see Fig. 6e). This relates to the preliminary phase of the research work, not yet translated into clinical practice, where model stability and security are more relevant.

In terms of ‘Technical validation’, the stability of AI models appeared to be adequately addressed by only a moderate proportion of TG responders (58%), mainly using sensitivity analysis techniques (35%), as shown in Fig. D2 in Supplemental File 4, illustrating the lack of awareness of the importance of this type of analysis. Nested cross-validation was considered by most TG respondents (73.3%) to be the most appropriate approach for training a model on a small dataset (see Fig. D3 in Supplemental File 4). The results show that the training and validation of AI models in a distributed environment is still not a common practice, as 50% of the TG respondents stated that they had no experience in this area. For the other half who were familiar with the topic, internal plus external validation was the only approach chosen.

In relation to the ‘Statistical analysis and evaluation metrics’, regression tasks appeared to be less popular among TG responders, as a modest share of responders (26.7%) declared that they did not use any relevant metrics. Those familiar with the problem mostly used the average error metrics and the 95% confidence interval, while only a small share considered bias and variance (16.7), as shown in Fig. D4 in Supplemental File 4.

With regard to ‘Bias and fairness’ issues, while TG respondents considered selection bias (as mentioned in the comparison section), they suggested that there may be constraints when taking necessary actions. Model checking against datasets having a real-world distribution of classes appeared to be mostly performed only ‘when it is possible’ (70%). Only a modest share of respondents (26.7%) declared that they always performed such a check. Similarly, fairness evaluation ‘when it is possible’ is reported by 57% of respondents, and the analysis of potential biases ‘when this information is available’ is reported by 77%.

### In-depth findings for the CG

In this subsection, we present the questionnaire results for the CG (19 respondents, with the following profile middle-aged clinicians aged between 40 and 60 years old (47.4%), working in public hospitals (52.6%), being relatively new to the AI field, *i.e.*, having between 1 and 5 years of experience with it (42.1%).

Regarding the ‘Overall strategy for model building and validation’, it can be observed (Fig. E1 in Supplemental File 5) that most clinicians lack specific knowledge on performing pre-checks on new data (42.1% responded “I don’t know”, followed by 36.8% who responded “Maybe, it depends on the use case and data availability”), model error analysis (80% mentioned “I do not know how to do it”), and robustness checks against adversarial examples (47.4% responded “I do not know how to do it”).

**Fig. 7:**
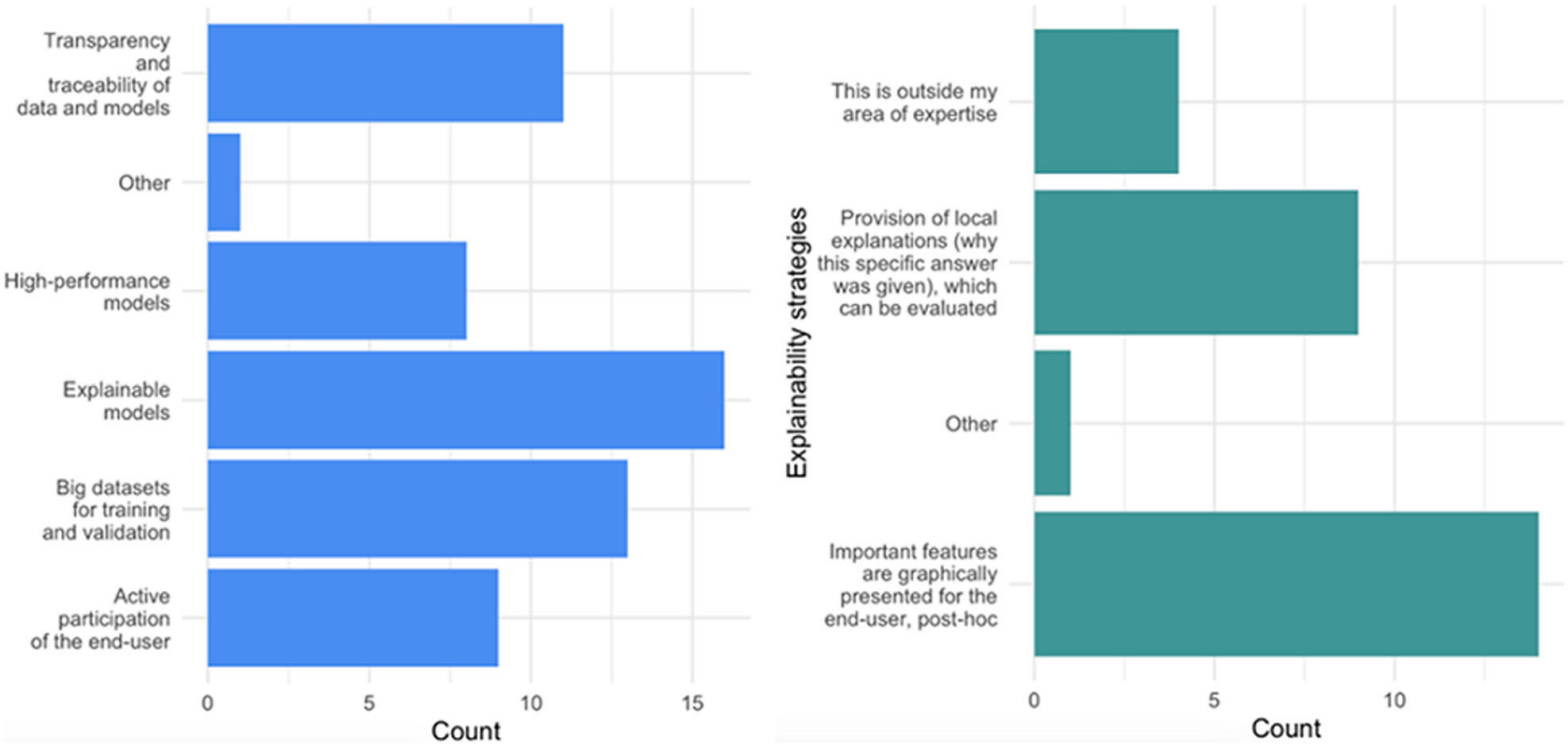
Trustworthiness factors and explainability strategies, from left to right

Interestingly, most clinicians consider explainability to be the most important trustworthiness factor, followed by large datasets for training and validation and transparency and traceability of data and models. Additionally, most CG participants consider a post-hoc graphical feature presentation as the preferable explainability strategy, followed by the provision of local explanations (Fig. 7).

Regarding ‘Technical validation’, unsurprisingly, it can be observed (Fig. E2 in Supplemental File 5) that most clinicians lack specific technical knowledge or experience in using nested cross-validation (63.2%) and distributed learning (63.2%). Most clinicians are also constrained in approaches performing validation with external datasets only when possible (52.6%), perhaps due to the lack of data availability, and in using only real data in validations (68.4%) (Fig. E3 in Supplemental File 5). CG seemed reluctant to use synthetic data in validations, with 21.1% using real and synthetic data after assessing the synthetic data validity, 5.3% using both indiscriminately, and 5.3% using only synthetic data. Additionally, most CG respondents do not rely on permutation-based techniques or find it out of their expertise, with a smaller percentage of CG using them—mostly target shuffling but also sensitivity analysis (Fig. E4 in Supplemental File 5).

Regarding ‘Statistical analysis and evaluation metrics’, it can be observed (Fig. E5 in Supplemental File 5) that most clinicians lack specific knowledge on comparing radiomics signatures to nomogram models (the majority of respondents, 52.6%), followed by 31.6% with “Sometimes, when clinical variables are available”). Only a smaller number (10.5%) of respondents answered that they always asked for clinical parameters.

Regarding ‘Bias and fairness’, the study found that despite understanding their importance in AI research, most clinicians conduct relevant validation tasks related to the real-world distribution of classes and fairness of the model for potential biases only when possible (63.2% and 42.1%, respectively). Despite this, the percentage of respondents who evaluate real class distribution and model fairness (21.1% and 31.6%, respectively) is higher than those who never do it (15.8% of “No, never” for both) (Fig. E6 in Supplemental File 5).

### Themes and qualitative analysis

Four themes were identified from the analysis of the open answers in the concluding section (Table E1 Supplemental File 5). The three themes (capability, utility, and adoption) follow the evaluation framework of TEHAI [12], and the fourth theme, trustworthiness, relates to one of the seven key requirements that AI systems should meet the criteria set in the ALTAI list [9]. Recurrent terms that emerged from the open answers were depicted in a ‘word cloud’ plot (Fig. E7 in Supplemental File 5). Each theme was elaborated with a verbatim response (Table E2 Supplemental File 5).

#### Theme 1: utility

This theme stresses the need for the AI system’s usefulness from a variety of perspectives, such as contextual relevance, safety, and ethical issues related to potential clinical applications. Moreover, it evaluates the system’s effectiveness as determined by the quality, adoption, and alignment metrics. The participants prioritised usability, clinical utility, clinical net benefit, clinical endpoints, biological plausibility, causality, machine learning models for prediction, pretreatment prediction and measures to reduce attention theft.

#### Theme 2: capability

This theme concentrates on the AI system’s inherent technological capacity to carry out its anticipated function by examining significant elements of the AI system’s development. This was a prominent theme evidenced by the participants’ emphasis on internal validation and clinical evaluation to estimate performance over time and identify potential concept drifts leading to a degradation in performance.

#### Theme 3: adoption

The adoption and integration of AI systems in healthcare delivery have been reported as problematic, even for those that have demonstrated efficacy. The system’s usability for end users and beneficiaries, as well as its ability to interoperate with external legacy IT systems inside and outside of the user-care facility, should be possible without any problems. This was highlighted as a priority factor to be evaluated when an AI system is being considered with an emphasis on ease of integration into clinical settings and cost effectiveness.

#### Theme 4: trustworthiness

This theme comprises two subthemes that are included in the well-established trustworthiness framework, which proposes a set of seven main requirements and a related assessment list that AI systems should satisfy to be regarded as trustworthy [9]:

- Explainability: participants believed that they should be provided with good insight into the reasons why an AI model gives a particular output or decision. To achieve explainability, AI models need design choices including the use of transparent models, feature importance analysis, and other techniques that can help to identify the factors that influence the output of the AI model [26];
- Traceability: to preserve a thorough record of the provenance of the data, procedures, and artefacts involved in the creation of an AI model, traceability is seen as a crucial prerequisite among the participants for trustworthy AI.

## Discussion

AI medical solutions aim to alleviate the burden of the exponentially growing numbers of medical scans and their impact on shortages of highly skilled healthcare professionals, enabling timely, precise diagnosis, classification, and treatment response prediction.

Large EU-funded research projects developing different AI-powered tools for cancer imaging involve healthcare professionals and AI research and development teams. Their collaboration and shared understanding of validation strategies are crucial for the development of safe AI solutions for clinical practice. Validation of these tools is critical as it impacts the safety, trust and credibility of AI solutions for healthcare and medical imaging. The first EU regulatory framework for AI (the AI Act) was proposed by the European Commission in April 2021 and adopted by the European Parliament in March 2024 for setting obligations for providers and users based on different risk levels of AI systems [27]. Most imaging and diagnostic tools will be classified as high-risk having to comply with traceability, transparency and users’ surveillance [28]. The AI Act will further challenge the implementation of AI imaging applications while improving their trustworthiness patient safety and non-discriminatory characteristics. Ensuring that both clinical and technical experts understand and apply good AI validation practices, is a way to promote compliance with the AI Act and consequently to facilitate deployment of their AI applications.

This work addresses the question, ‘How is AI validated and reported currently in the health/cancer imaging domain by technical experts and clinical experts’, via the analysis of the responses to several focused questions. The comparison of responses of the two groups, technical and clinical, revealed some points for further consideration toward more standardised and effective AI validation.

The data analysed originate mostly from researchers in public institutes. Although most of the TG were generally younger than the CG, both groups were relatively new to the field of AI, ranging from 1 year to 5 years of experience. The aforementioned fact and the fact that validation strategies often come at later stages for several projects contribute to several ‘not my area of expertise’ answers. In the same vein, work in projects of limited duration toward prototyping AI solutions, rather than deploying AI in clinical practice, influences some of the answers “if time allows” in various validation-related questions.

The two groups have different understandings and priorities regarding validation factors related to trustworthy AI models. The TG prioritises transparency and traceability of data and models, more than CG, potentially due to CG’s less exposure to AI development processes and the importance of the different dataset and model quality parameters on AI trustworthiness. On the other hand, CG focuses more on the explainability of AI models. As suggested by Liu et al [29], when considering physicians, explainable AI may seriously influence AI technology trust and perceived value, with a significant impact on the intention to use AI. It would be advisable that both groups share a common understanding and appreciation of trustworthy AI. In fact, Jacobson and Krupinski [30] recommended the involvement of domain experts such as radiologists in the development process to facilitate early interaction and ensure that requirements are aligned with clinical practice. The work of Cai et al [31] provides insights into the onboarding needs of medical practitioners regarding human-AI collaborative decision-making. They reveal that medical practitioners require upfront information about the global properties of the models, including their strengths, limitations, point-of-view, and overall design objective, beyond just understanding the case-specific reasoning behind model decisions.

Overall, some deviations in the responses provided by the two groups, for example, with respect to ‘cross-validation’, could be explained by their technical nature, as well as the lack of common understanding between the two groups regarding the AI development stages. Even when the most voted option was the same, *e.g.*, regarding segmentation evaluation metrics, the percentage was different in the two groups (*e.g.*, 80% *versus* 47.3%), and CG considered several questions ‘not my area of expertise’, being more on the technical side. The lack of widely accepted standardised methods in AI technical validation and reporting approaches hinders widespread adoption and integration into routine care. A study by Chen et al [32] on the adoption of clinical AI by physicians and medical students revealed that despite awareness of AI’s increasing use, only 10–15% of physicians and medical students have practical experience and knowledge about AI. Common understanding would be required as regards the metrics to validate segmentation models for use in clinical routine, or the usefulness of an AI model against a clinical variables’ nomogram. Since clinicians are used to employing nomograms for disease prognosis, it could be a natural extension to compare clinical variable-based nomograms with radiomics signatures for the validation of AI models.

It is also worth noting that the CG group is more reserved for using synthetic data. While the potential of synthetic data in clinical research has been reported [33, 34], it has also been recognised that more research toward wider adoption is needed, including among others, the cost-effectiveness of generating synthetic data and assessment of disclosure risk.

With the TG in focus, the responses gave rise to some concerns regarding the consistency of approaches when checking the stability and robustness of AI models for adoption in clinical practice. This finding aligns with the research gaps identified by Davis et al [35], emphasising the need for working on the AI modelling lifecycle, in maintenance, performance monitoring, and updating. This quantitative finding is also in line with the ‘Capability’ theme highlighted from the qualitative analysis of the open questions, as regards the identified need to estimate model performance over time and identify potential concept drifts.

The fact that most TG respondents declared analysing the reasons behind the misbehaviour of their AI model ‘only if time allows’ may need further consideration. This may relate to the fact that, in the context of the AI4HI projects, the respondents are involved in the development of research prototypes that are not expected to be clinically deployed immediately after their delivery. Evidently, promoting error analyses as a standard part of AI validation could enhance traceability and trustworthiness, and thus deployment of AI.

In addition, the TG acknowledged fairness issues (50%) but less competence in addressing them, suggesting a lack of thorough examination of real-world data bias in practice. This highlights the need for further investigation and consideration when real-world deployment of research AI prototypes is intended. Bias issues are expected to be better dealt with when more extended datasets are available, either in centralised or federated repositories, which is a direction toward which the research community is moving.

The study reveals a diverse strategy for model validation in the TG, with respondents choosing various options such as external and internal validation, large and small test samples, and independent research teams. While this diversity of opinion is partly explained by the diversity of conditions, options, and validation tasks in the various AI4HI projects, it also suggests the lack of a standardised, universally accepted procedure. Access to reliable data sets for independent validation by the original group is challenging in the medical field, whereas “external validation using new test data done by an independent research team” is more common in classical clinical research. As highlighted in a recent review [4], randomised controlled trials on AI in clinical practice are scarce, while the small sample size and single-centre design hinder the model's generalizability. Recently proposed validation platforms [36] aim to use real-world data from clinical data warehouses for external AI validation and method comparison/benchmarking.

The qualitative analysis aimed to give respondents more freedom in expressing their own needs in relation to the clinical validation of AI models. This analysis revealed four priorities: utility, capability, adoption, and trustworthiness, the importance of which and their role in supporting the integration of AI into clinical workflows have been well described in the literature. These four themes are linked to the quantitative analysis of the closed questions. For example, regarding ‘trustworthiness’, the quantitative analysis also identified explainability and traceability as the most frequently selected options for the trustworthiness of the model for the CG and TG, respectively. They are also documented as criteria in the TEHAI [12, 13, 15] evaluation framework.

Overall, this work, highlighting similarities and differences in the perception of AI validation tasks by technical and clinical experts involved in AI for health/cancer imaging, emphasises the need for more standardised and widely accepted AI validation methods to bridge the gap between technical and clinical perspectives and for a comprehensive understanding and planning of AI validation and AI trust.

A limitation of this work is the low number of participants and the research nature of the AI development work in which most of the respondents are involved, which also introduces a slight imbalance between TG/CG responses. Responses may have been different if they were coming from technical and clinical experts working on or with deployable AI at more advanced stages of AI development and not research prototypes. The term validation is not used consistently in the literature, and among different groups, as suggested by [37]. Responses may also differ based on a possible different understanding of the AI validation definition from technical and clinical respondents—while the former see model validation as a technical step of hyperparameter tuning to improve model performance, the latter see it as a process for implementation in the clinical setting using one or more realistic and external test sets. A survey with wider coverage with participants representing in a more balanced way various workplace contexts, more advanced AI development stages, age, years of experience, and background profiles could shed more light on these questions and extend the identification and understanding of possible further recurring paradigms and attitudes among groups. Of note, in this work, the teams are characterised in a binary manner as technical or clinical, based on their main role in research projects, while this does not exclude the existence of both areas of expertise within the team, a feature that may become more evident in the future, as research and clinical practice require more interdisciplinarity and would suggest the addition of a third class of responders (the mixed group).

In conclusion, this work contributes toward the definition of best practices in cancer imaging AI validation, to cover both technical and clinical perspectives while also considering AI trustworthiness. The findings highlight the need for attention and elaboration on issues that are now weakly or controversially addressed. Within the AI4HI cluster, there is a continuous effort toward exploring common practices and sharing knowledge and experience, including the harmonisation of validation methodologies and reporting guidelines/tools for trustworthy AI in cancer imaging. The work revolves around AI in cancer imaging and capitalises on the accumulated researchers’ experience in this area about: (a) the opinions/expectations of radiologists and AI developers; (b) the clinical challenges to be solved by AI methods, *e.g.*, segmentation of lesions, diagnosis, and prognosis of metastasis; (c) the particularities of data sources; and (d) frequent experiences in validation. The findings are extensible to the wider health imaging domain and other health data-driven areas, bearing in mind that the abundance of data for validation may vary among domains, as well as the complexity of problems and expectations for performance.

## Supplementary information

**Additional file 1: Table C 1**. The correspondence of ‘Roles’ Combinations to Clinical and Technical group. **Table C 2**. Age-working place combinations of the whole group. **Figure C 1**. Responses regarding user roles (model training, model validation (technical), model validation (clinical), clinical expert, data provider and data curation). Multiple responses were possible. **Figure C 2**. Stacked bar plots on some ‘Technical validation’ topics comparing technical and clinical profiles points of view. **Figure C 3**. Stacked bar plots on some ‘Bias and fairness’ topics comparing technical and clinical profiles points of view. **Figure D 2**. Bar plot about the use of permutation-based techniques to assess model stability by TG respondents. The x axis indicates the proportion of responses. **Figure D 3**. Bar plot about the use of nested cross-validation by TG respondents. The x axis indicates the proportion of responses. **Figure D 4**. Bar plot about the strategy of reporting regression models adopted by TG re-spondents. The x axis indicates the proportion of responses. **Figure D 5**. Dataset Splits. **Figure D 6**. Use of Permutation Techniques. **Figure D 7**. Segmentation evaluation metrics. **Figure D 8**. Regression evaluation metrics. **Figure D 9** Reporting on regression models. **Figure E 1**. Stacked bar plots on the ‘Overall strategy for model building and validation’ top-ics where clinicians would benefit from further exposure. **Figure E 2**. Stacked bar plots on the ‘Technical Validation’ topics where clinicians would ben-efit from further exposure. **Figure E 3**. Stacked bar plots on the ‘Technical Validation’ topics where clinicians are some-how limited. **Figure E 4**. Bar plots on the multiple-choice question regarding permutation-based tech-niques. **Figure E 5**. Stacked bar plots on the ‘Statistical analysis and evaluation metrics’ topics where clinicians would benefit from further exposure. **Figure E 6**. Stacked bar plots on the ‘Bias and Fairness’ topics where clinicians are some-how limited. **Figure E 7**. Word cloud from common terms emerged from open questions on AI validation strategies. **Table E 1** List of themes that emerged from the data analysis and concepts per theme. **Table E 2** List of themes that emerged from the data analysis and concepts per theme. In the verbatim quotes, ‘P’ stands for participant

## Data Availability

All data analysed during this study are included in this published article and its supplementary information files.

## Abbreviations

AI: Artificial intelligence
AI4HI: AI for health imaging
ALTAI: Assessment list for trustworthy AI
CG: Clinical group
TEHAI: Translational evaluation of healthcare AI
TG: Technical group
