# Supplementary material for: Differences in technical and clinical perspectives on AI validation in cancer imaging: mind the gap!
Source: Eur Radiol Exp. 2025 Jan 15;9:7. doi: 10.1186/s41747-024-00543-0 (PMC11735720; doi:10.1186/s41747-024-00543-0)
Supplement: Supplementary file 1 — Additional file 1: Table C 1. The correspondence of ‘Roles’ Combinations to Clinical and Technical group. Table C 2. Age-working place combinations of the whole group. Figure C 1. Responses regarding user roles (model training, model validation (technical), model validation (clinical), clinical expert, data provider and data curation). Multiple responses were possible. Figure C 2. Stacked bar plots on some ‘Technical validation’ topics comparing technical and clinical profiles points of view. Figure C 3. Stacked bar plots on some ‘Bias and fairness’ topics comparing technical and clinical profiles points of view. Figure D 2. Bar plot about the use of permutation-based techniques to assess model stability by TG respondents. The x axis indicates the proportion of responses. Figure D 3. Bar plot about the use of nested cross-validation by TG respondents. The x axis indicates the proportion of responses. Figure D 4. Bar plot about the strategy of reporting regression models adopted by TG re-spondents. The x axis indicates the proportion of responses. Figure D 5. Dataset Splits. Figure D 6. Use of Permutation Techniques. Figure D 7. Segmentation evaluation metrics. Figure D 8. Regression evaluation metrics. Figure D 9 Reporting on regression models. Figure E 1. Stacked bar plots on the ‘Overall strategy for model building and validation’ top-ics where clinicians would benefit from further exposure. Figure E 2. Stacked bar plots on the ‘Technical Validation’ topics where clinicians would ben-efit from further exposure. Figure E 3. Stacked bar plots on the ‘Technical Validation’ topics where clinicians are some-how limited. Figure E 4. Bar plots on the multiple-choice question regarding permutation-based tech-niques. Figure E 5. Stacked bar plots on the ‘Statistical analysis and evaluation metrics’ topics where clinicians would benefit from further exposure. Figure E 6. Stacked bar plots on the ‘Bias and Fairness’ topics where clinicians are some-how limited. Figure E 7. W [file 41747_2024_543_MOESM1_ESM.pdf]

Differences in technical and clinical perspectives on AI validation in cancer imaging: mind the gap!
ELECTRONIC SUPPLEMENTARY MATERIAL

Supplemental File 1. Useful glossary

AI performance: Competence to measure the ability of an AI model to make correct predictions from data. Depending on the model type, different metrics can be selected to evaluate its performance. The context and the end goal of your AI model are also important in choosing the best approach to quantify performance.

AI explainability: Strategy to provide the end-user an understanding and increased trust in the decisions made by complex and non-transparent AI models. This can involve the use of graphical representations of relevant features or the presentation of local explanations through visualization maps. As described by Champendal et al [21], 'XAI techniques provide justifications, explanations and insights into how AI models arrive at specific decisions, with the objective to instill trust, transparency, and confidence in the system'.

AI interpretability: A term often used interchangeably with AI explainability. It can be distinguished as the mechanism by which the end-user must be able to predict the model's decisions by changing the input data or model parameters, without requiring additional techniques to understand these decisions. As described by Champendal et al [21], 'Interpretable AI focuses on designing transparent models that offer human-readable representation of their decision-making process'.

AI uncertainty: The uncertainty is based on how sure the model is about its output.

AI generalisability: The stability of the performance and robustness of a model to data unseen during training. For example, a cancer detection model is well generalizable if the performance is similar across different centers and domains (scanner vendor and imaging protocol).

Ground truth: The term used in Machine Learning to describe gold standard, expert annotation corresponding to the data. For example, delineation of a lesion in an MRI or biopsy proven diagnosis are used as the ground truth to train AI models.

k-fold cross validation: technique commonly used for model validation and hyperparameter tuning. The dataset selected for model training and validation is split into k different subsets of equal size, along k iterations, the model is trained using k-1 subset and validated with the remaining subset. The prediction error or accuracy is computed separately on each fold. From a good model we would expect a high mean accuracy in the prediction and a low variance (good performance independently of the subset used for training/validation). The methodology is sometimes also used for models' hyperparameters selection.

Nested cross-validation: this process reduces the bias introduced by a simple k-fold cross validation used for both hyperparameters selection and models validation. It includes two different loops, an inner loop, and an outer loop. The data is split into k subsets. Then, on each iteration, a new k-fold cross validation is conducted for hyperparameters tuning (inner loop). Once the hyperparameters are selected, the error/accuracy of the final model is computed with the remaining subset of the outer loop.

Permutation-based techniques: class of methods used to verify the statistical significance and stability of the performances of a machine learning or a statistical method.

Target shuffling: a technique used for testing the statistical accuracy of a machine learning model, confirming that the model is performing well not by chance but because it has discovered real correlations. It consists in permuting the values of the target variable(s) and training a new model several times. The performance of the new models is expected to drastically drop, as the shuffling operation would break any relationships between the input and target variables.

Sensitivity analysis: class of techniques used to assess the robustness, i.e., 'sensitivity' of the results of a trained machine learning model when changes are applied to the values of the model's input variables or the hypotheses at the base of the model development.

External validation: this refers to a completely independent dataset, not used in any manner during model creation and optimization, and potentially produced in an independent manner, for example in a different site than the ones from which the training data originate.

AUC: the area under the ROC curve, where ROC (Receiver operating characteristic curve) is a graph that shows the relation between sensitivity and specificity in different classification setups (e.g., thresholds). AUC provides a summary of the ROC curve, and when close to it suggests a perfect classifier.
CI is the confidence Interval.

Nomogram. A graphical representation of the relation among variables, which allows to easily estimate one, if the others are known. Used in clinical practice, among others.

Radiomics. Quantitative characteristics extracted from medical images (in 2D or 3D), expressing for example texture, morphology, etc. Useful also as features in machine learning.

DICE: measures the similarity between the actual object (ground truth) and the object generated by the segmentation method under consideration

Supplemental file 2. The questionnaire

AI Validation working group AI4HI Survey

Section 1/7 Profile

1. **Which is your Role (or Roles) in an AI project?** *

Model Training

Model Validation (Technical)

Model Validation (Clinical)

Data Curation

Data Provider

Clinical expert

2. **Years of expertise in the domain**

1-5

5-10

10-20

above 20

3. **In which continent are you based? ***

Europe

North America

South America

Africa

Asia

Oceania

4. **Do you work in one of the AI4HI cluster projects?***

Yes

No

5. **Do you work in***

A public research institute

A private company

A private or public health organisation

6. **Age range ***

20-40

40-60

above 60

<u>Section 2/7 Overall Strategy for model building and validation</u>

7. **In model building, which balance do you consider between interpretability and performance***

I prefer to try maximizing performance with a black box ML model

I look for a balance between performance and interpretability

I focus mainly on the interpretation of the models without caring if there is a performance decrease

This is outside my area of expertise

8. **How important is biological validation when you are interested in deploying a model for clinical use? ***

The term "biological validation" elaborates the association between radiomics phenotypes and the underlying tissue biology

Less Important

1 2 3 4 5 More Important

9. **Do you always use ground truth in your validation studies? ***

Yes, I do validate my models only when ground truth is available

No, I also use unsupervised methods to provide validation scores in new unlabeled data

I don't know

10. **Which factors are important for the end users to consider models as trustworthy? (Choose all that apply)**

Big datasets for training and validation

Explainable models

High-performance models

Active participation of the end-user

Transparency and traceability of data and models

I don’t know

Other…

11. **What is your strategy regarding models' explainability? (Choose all that apply) ***

Important features are graphically presented for the end-user, post-hoc

Provision of local explanations (why this specific answer was given), which can be evaluated

No need for explainability

This is outside my area of expertise

Other

12. What is your strategy towards improving generalizability of your models? *

My strategy is to push the models, learn invariant features that will generalize well by means of multi-institutional heterogeneous data

All I care about is to have robust and reproducible models with data coming from my institution

My primary focus is my institutional data and performance in such data, however I use a single or limited external dataset to understand if my model generalizes well

This is outside my area of expertise

Other…

13. Do you perform pre-checks on new data to assess limitations of your models' applicability? *

No, the model can always be used without limitations - pre-checks are not necessary

Yes, I always perform some pre-checks to test if the next unknown case is an inlier or outlier to the training dataset to accept or refuse the request to use the model

Maybe, it depends on the use case and the availability of new data

I don't know

14. Do you perform model error analysis? *

Yes, always by identifying sub-cohorts of patients with a significant reduction in the performance, informing the end-users

As above answer plus taking actions to correct for the above-mentioned problems

Only if time allows

No, never

I do not know how to do it

Other …

15. Do you analyze the reasons behind wrongly classified data?*

Yes, always

Yes, only when it is possible

No, never

I do not know how to do it

16. Do you check the robustness of your ML models against adversarial examples?*

Yes, always

Only if time allows

Never

I do not know how to do it

Section 3/7 Technical Validation

17. When you have a single small dataset (<200 patients) how do you split your data?*

Single split (80% for training/validation and 20% for testing)

Repeated split by changing the random seed-producing n different training/test splits

k fold cross-validation on the whole dataset

k fold cross-validation on 80% of the data and 20% of the data for testing

Other …

18. Which permutation-based techniques are you using?*

Sensitivity analysis

Target shuffling

I don't use any permutation-based techniques

Other …

19. In the presence of a small dataset (<200 patients) do you use nested cross-validation to select your Machine Learning Models?*

Yes, always

No, never

I don't know what that is

20. By which methods do you evaluate your models? (Choose all that apply)*

Numerical Evaluation (performance metrics)

Visual Analytics (Integrating expert knowledge through interactive visualizations, leading to semi-automatic validation)

Manual checks

Other …

21. If your model is trained in a distributed learning setting, which validation strategy do you opt for?*

Internal validation

Internal + External validation

External Validation only

I currently don't have experience with distributed learning

Other …

22. Do you use validation with external datasets - besides cross-validation?*

Yes, always

Yes, only when it is possible

No, never

23. Do you use both real and simulated/synthetic data in validations?*

Yes, I use both indiscriminately

Yes, I use both after assessing the validity of the synthetic data

No, I only use real data in validations

No, I only use simulated data in validations

Section 4/7 Statistical Analysis and Evaluation Metrics

24. How do you report the results of your binary classification model validation?*

The average AUC

The average AUC + 95%CIs

The average performance metric that is suitable for my specific use case

The average performance metric that is suitable for my specific use case with the corresponding 95%Cis

25. Which metrics do you use to validate segmentation models? (Choose all that apply)*

Only DICE Coefficient

DICE coefficient and other metrics related to mask overlapping between the predicted mask and ground truth (e.g., Intersection Over Union)

Volume-based metrics (e.g., relative volume difference, volume correlation)

Distance-based metrics (e.g., MSD, ASSD, etc.)

This is outside my area of expertise

Other …

26. **Which metrics do you use to validate your regression models? (Choose all that apply)***

The Mean Absolute Error (MAE)

The Mean Square Error (MSE)

The R-squared (R2) metric

Others

This is outside my area of expertise

27. **How do you report the results of your regression models?***

The error metric alone

The error metrics and the bias

The average error metric

The average error metric and 95% CI

The average error metric, 95% CI, the bias and the variance

None of the above

28. **Do you compare the usefulness of your imaging Machine Learning or radi-omics model against nomogram models based only on clinical variables?***

Yes, I always ask for having also clinical parameters

No, never

Sometimes, when clinical variables are available

Not under my area of expertise

Section 5/7 Bias & Fairness

29. At an initial phase of the project do you consider possible selection bias to your datasets?*

I’m aware of it, but I can't do anything about it

I’m aware of it and I’m using methods to detect and acknowledge it to the end-user

I’m aware of it and I’m using methods to mitigate

I don't know what selection bias is / I do not think that it is important

30. Do you evaluate/test using a set that represents a real-world distribution of classes (e.g., malignant tumor cases are much less than benign)? *

Yes, always

Yes, only when it is possible

No, never

31. Do you analyze your data and report for potential biases/skewness ?*

Yes, always

Yes, when this information is available

No, never

I do not know how to do it / I do not think that it is important

32. Do you evaluate/verify the fairness of your model for demographic, geo-graphic, ethnicity, age, sex, and socioeconomic biases? *

Yes, always

Yes, only when it is possible

No, never

I do not know how to do it / I do not think that it is important

33. **Which of the following potential biases do you consider necessary to be reported? (Choose all that apply) ***

Demographic

Geographic

Ethnic

Age

Sex

Socioeconomic

Health status related

None

Other…

Section 6/7 Concluding Questions

34. **Which of the following strategies do you use to validate your AI models (please indicate the ones that are most frequently applied in your projects) ***

Attempting several validations (internal or external) and reporting only the one that "worked"

Reporting apparent performance on the training dataset (no validation)

Reporting predictive accuracy on an undersized independent test sample

Internal validation using data-splitting where at least one of the training and test samples is not huge and the investigator is not aware of the arbitrariness of variable selection done on a single sample

Strong internal validation using 100 repeats of 10-fold cross-validation or several hundred bootstrap resamples, repeating all analysis steps

External validation on a large test sample, done by the original research team

Re-analysis by an independent research team using strong internal validation of the original dataset

External validation using new test data, done by an independent research team

External validation using new test data generated using different instruments/technology,

done by an independent research team

Other…

35. Other than the AI-validation related topics above, are there other AI validation perspectives, especially from a clinical point of view, that you think are important for an AI validation plan to cover?*

Open answer:

Section 7/7 Feedback about this survey

Please add below any comments or feedback you have about this survey.

If you wish to be notified about the results of this survey, please add below the email in which we can contact you.

It is absolutely optional, and only for the specific purpose of disseminating this survey's results.

Supplemental File 3. Details on the Comparison between the Technical and Clinical Group

Table C 1. The correspondence of 'Roles' Combinations to Clinical and Technical group

Clinical Group	Technical Group
"Model Validation (Clinical);Clinical expert"<br>"Model Validation (Clinical);Data Curation;Clinical expert"<br>"Model Validation (Clinical);Data Provider"<br>"Model Validation (Clinical);Data Provider;Clinical expert"<br>"Model Validation (Technical);Model Validation (Clinical);Data Curation;Data Provider;Clinical expert"<br>"Clinical expert"<br>"Data Provider"<br>"Data Curation"<br>"Model Validation (Clinical)"	"Model Training"<br>"Model Validation (Technical)"<br>"Model Training;Model Validation (Technical)"<br>"Model Training;Model Validation (Technical);Data Curation"<br>"Model Training;Model Validation (Technical);Data Curation;Data Provider"<br>"Model Training;Model Validation (Clinical);Data Provider"

Table C 2. Age-working place combinations of the whole group

Age/working place	20-40	40-60	above 60
public research institute	40.81	24.489	4.08
private company	4.081	4.08	0
private or public health organization	14.28	6.12	2.04

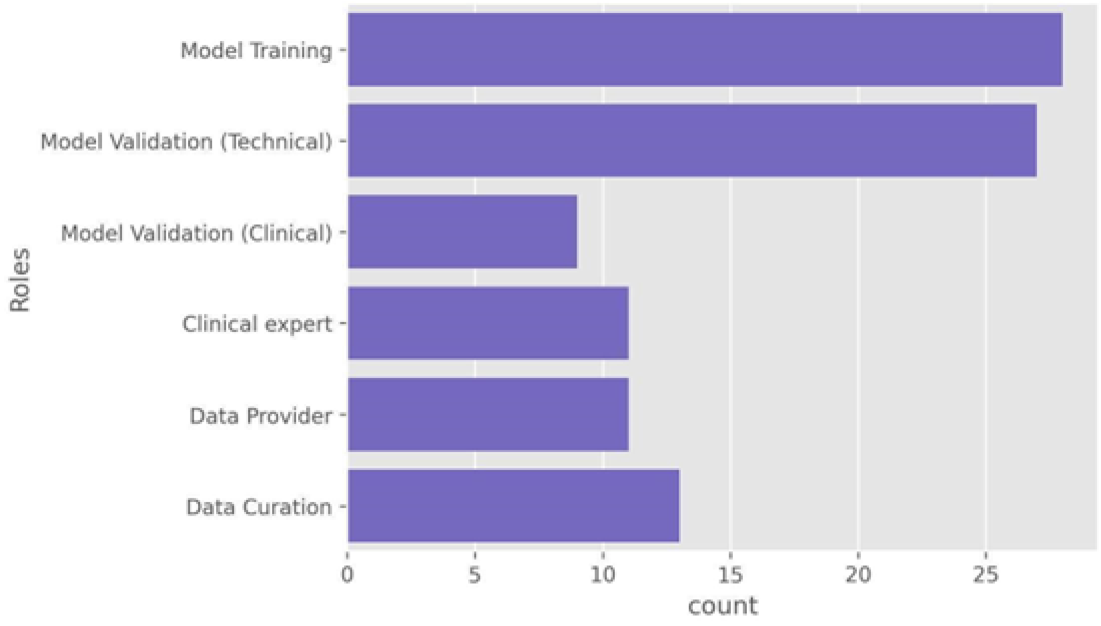

Figure C 1. Responses regarding user roles (model training, model validation (technical), model validation (clinical), clinical expert, data provider and data curation). Multiple responses were possible.

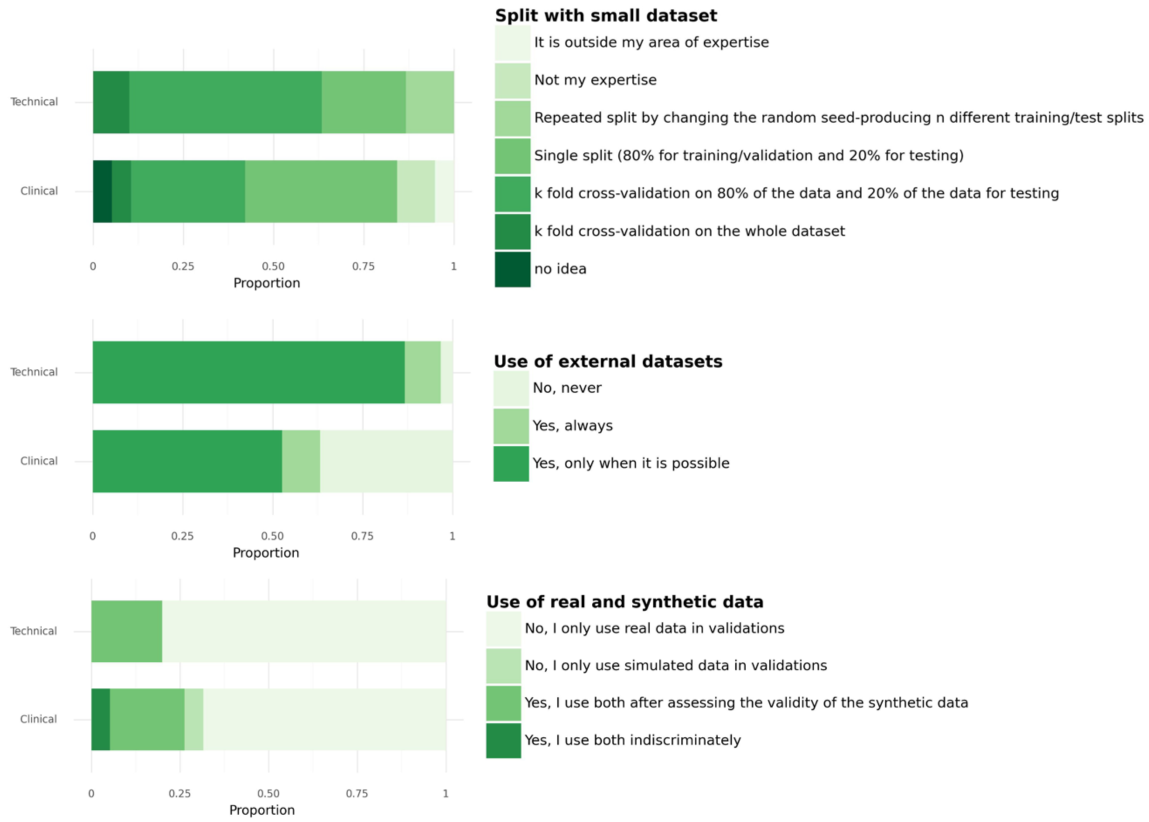

Figure C 2. Stacked bar plots on some 'Technical validation' topics comparing technical and clinical profiles points of view.

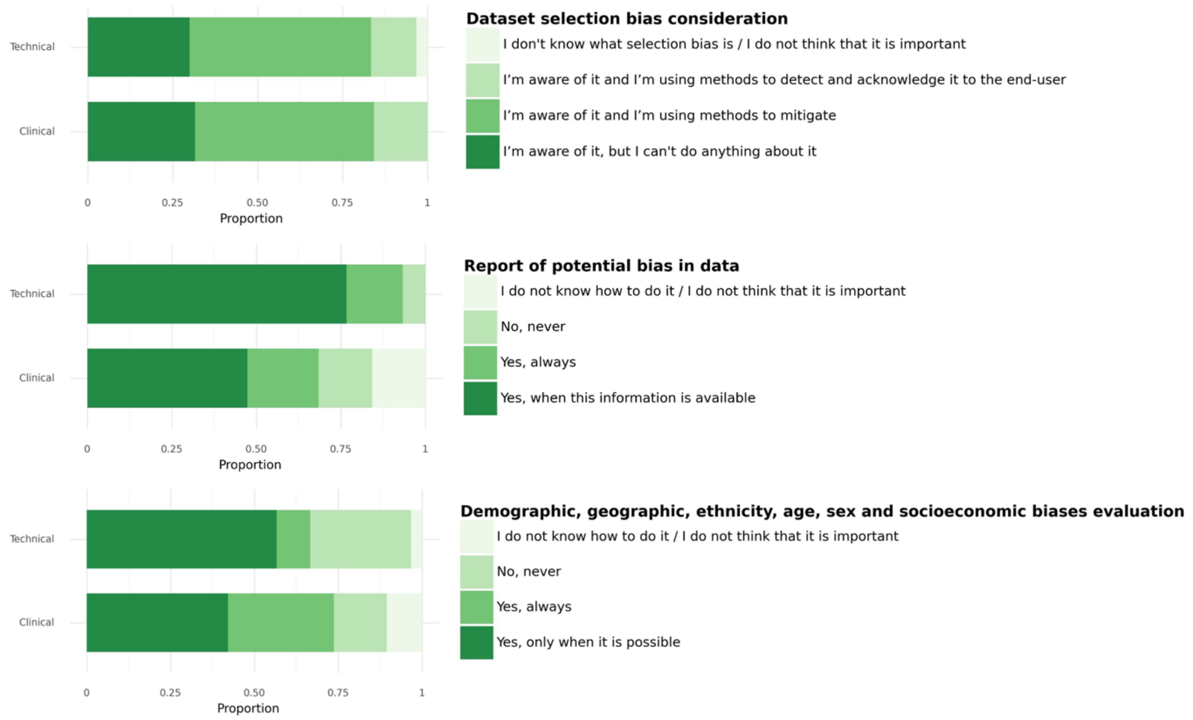

Figure C 3. Stacked bar plots on some 'Bias and fairness' topics comparing technical and clinical profiles points of view.

Supplemental File 4. Technical responders’ analysis details

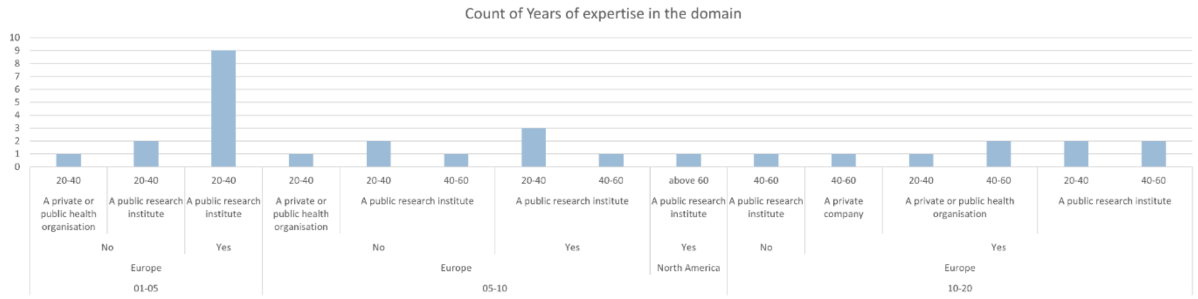

Figure D 1. Age-work distribution in the technical group

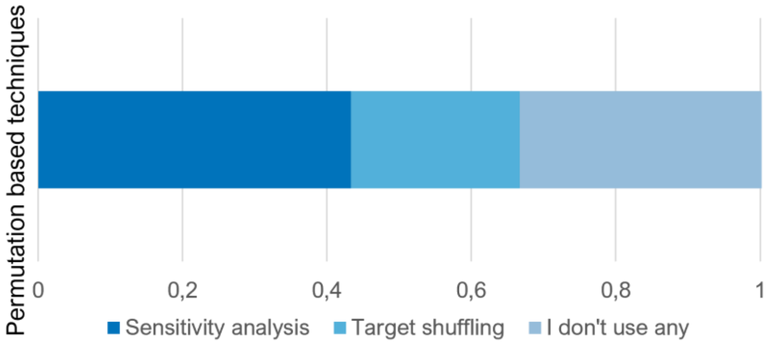

Figure D 2. Bar plot about the use of permutation-based techniques to assess model stability by TG respondents. The x axis indicates the proportion of responses.

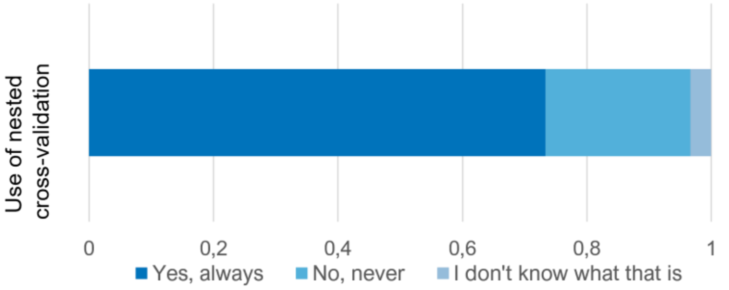

Figure D 3. Bar plot about the use of nested cross-validation by TG respondents. The x axis indicates the proportion of responses.

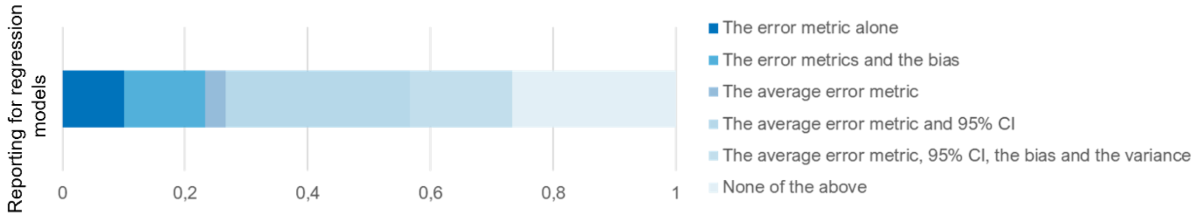

Figure D 4. Bar plot about the strategy of reporting regression models adopted by TG respondents. The x axis indicates the proportion of responses.

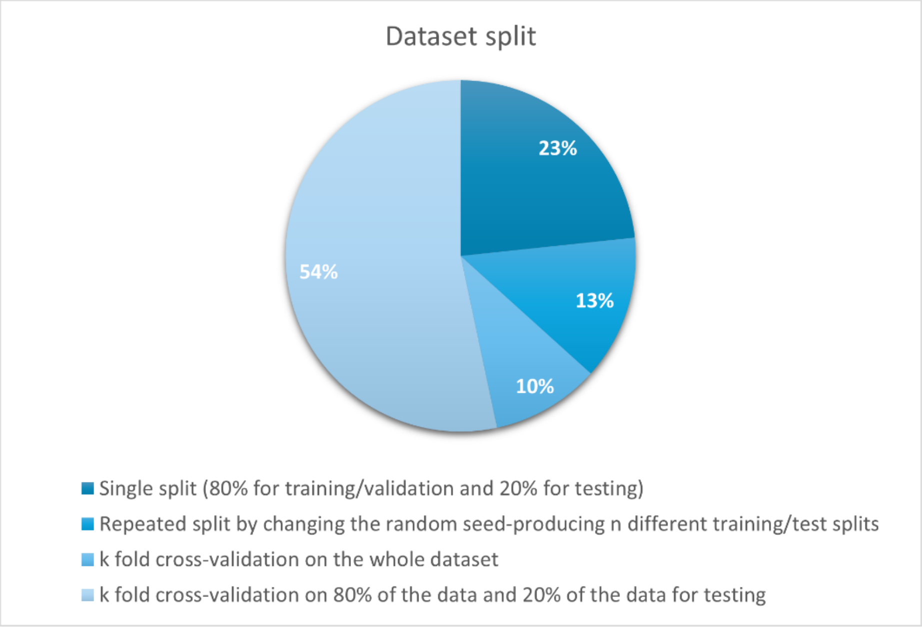

Figure D 5. Dataset Splits

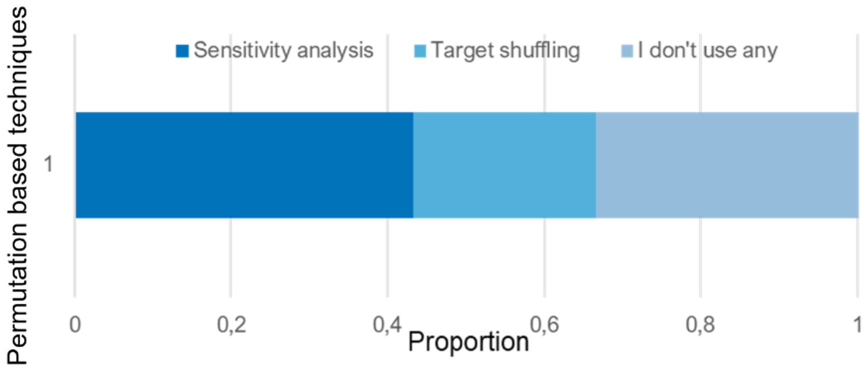

Figure D 6. Use of Permutation Techniques

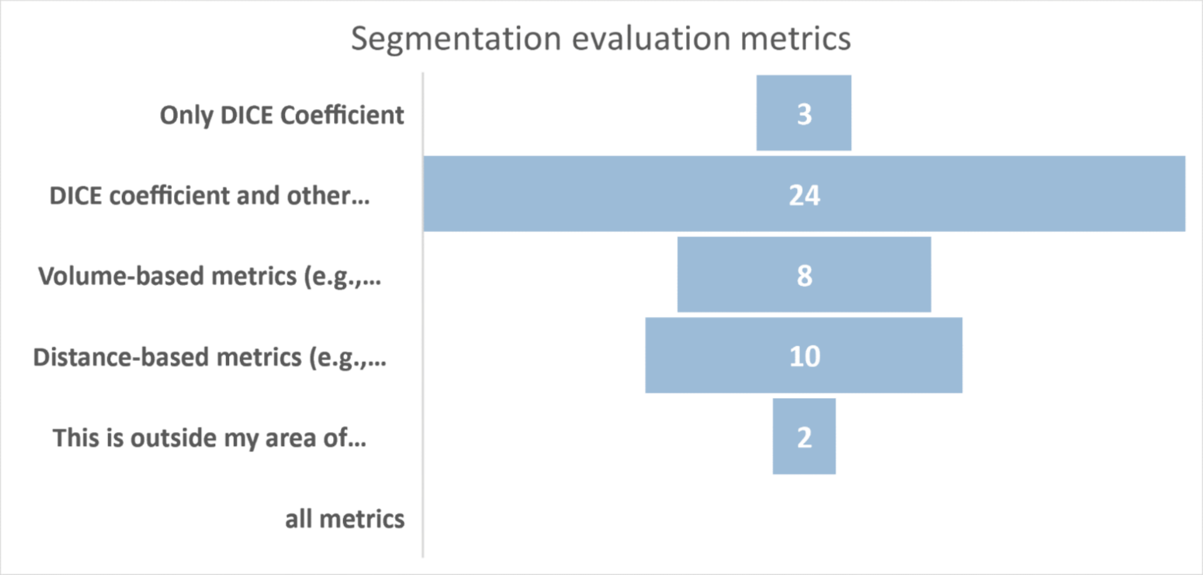

Figure D 7*. Segmentation evaluation metrics*

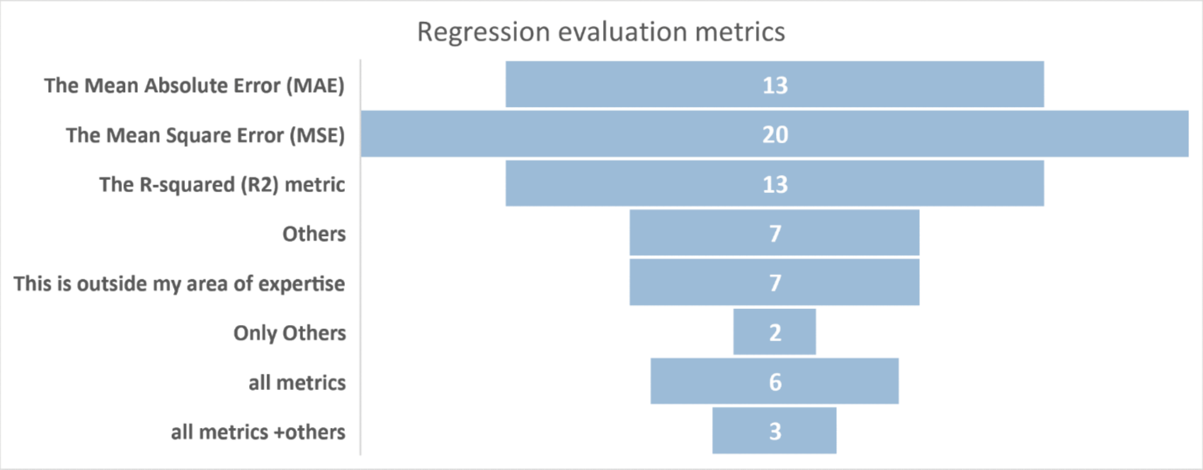

Figure D 8*. Regression evaluation metrics*

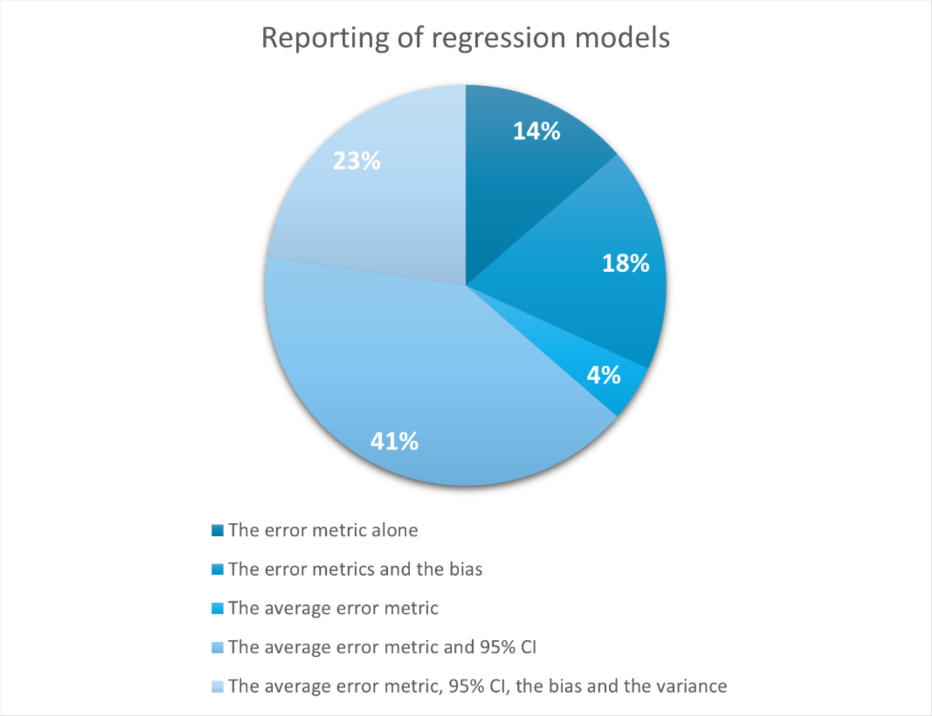

Figure D 9 Reporting on regression models

Supplemental File 5. Clinical Analysis Details and Qualitative Analysis

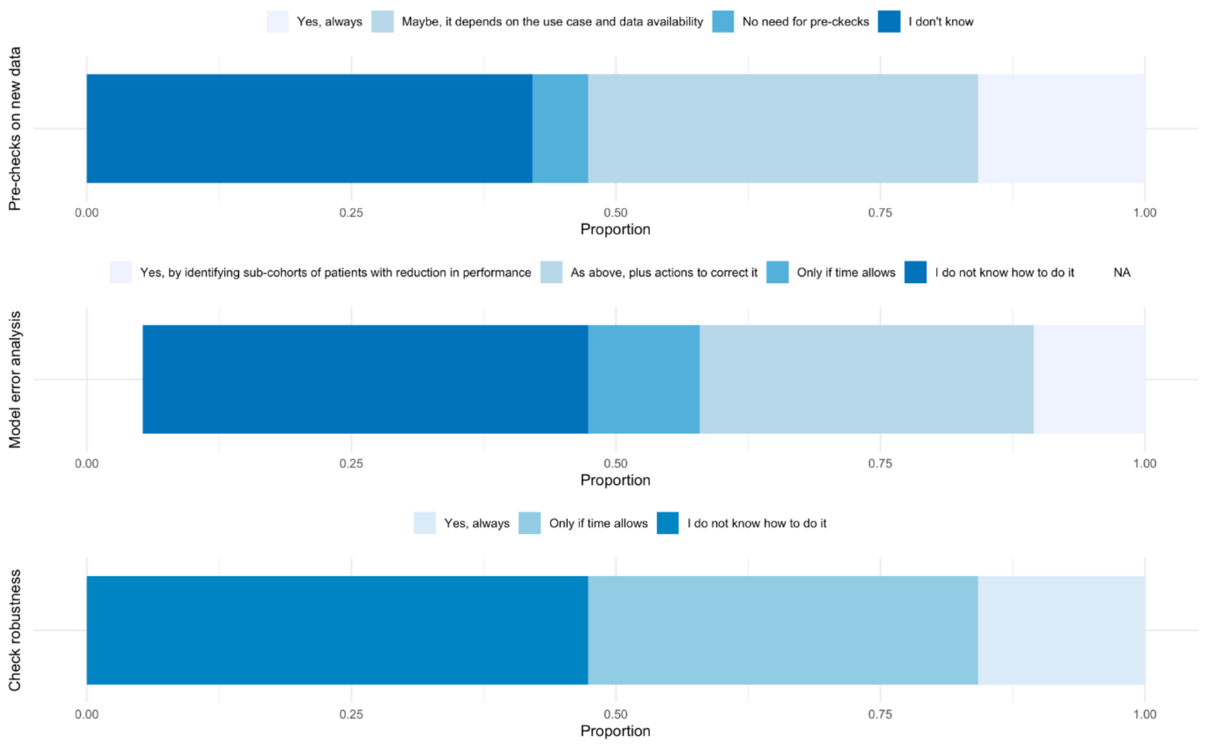

Figure E 1. Stacked bar plots on the 'Overall strategy for model building and validation' topics where clinicians would benefit from further exposure.

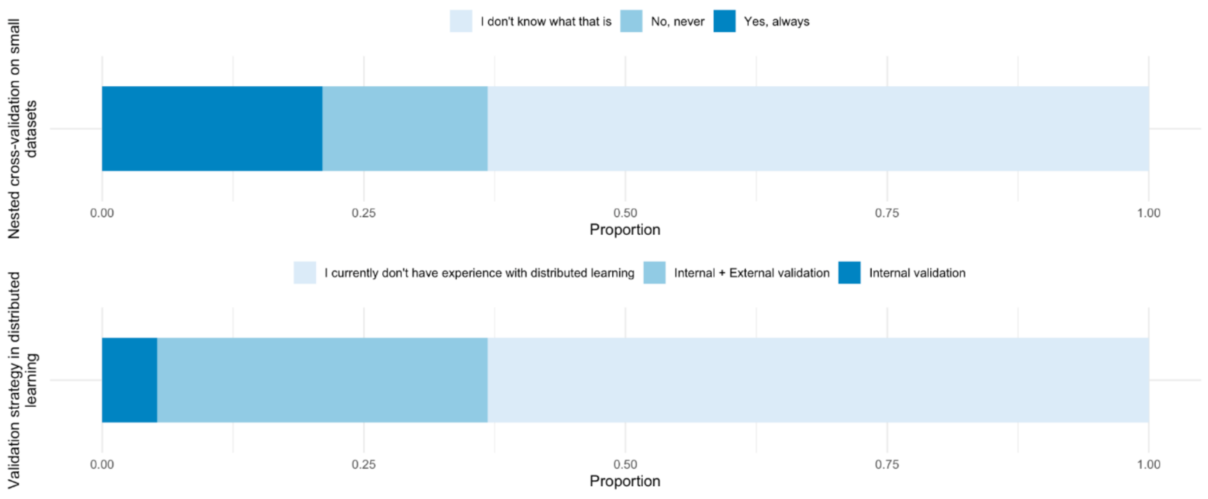

Figure E 2. Stacked bar plots on the 'Technical Validation' topics where clinicians would benefit from further exposure.

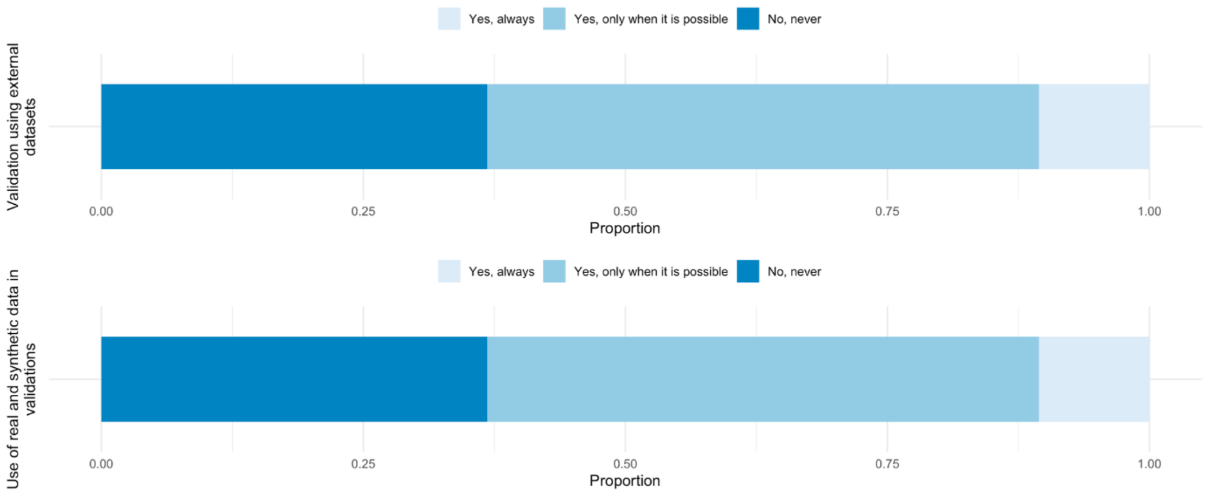

Figure E 3. Stacked bar plots on the 'Technical Validation' topics where clinicians are somehow limited.

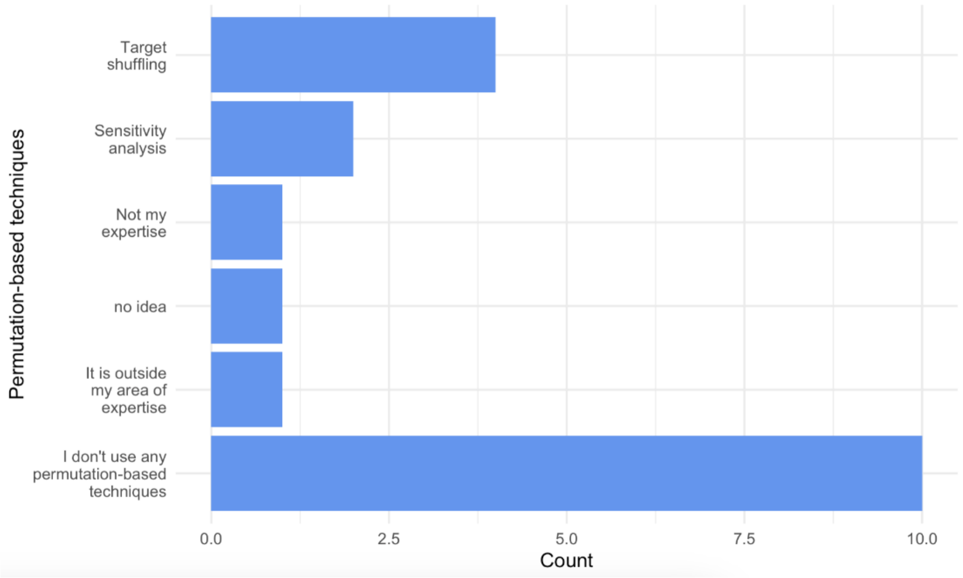

Figure E 4. Bar plots on the multiple-choice question regarding permutation-based techniques

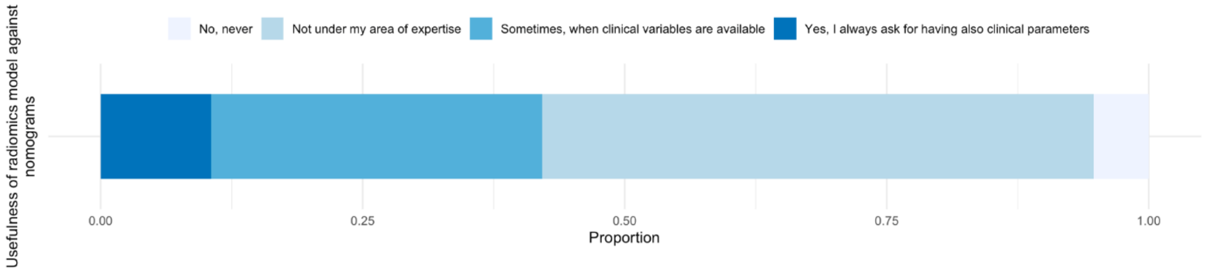

Figure E 5. *Stacked bar plots on the 'Statistical analysis and evaluation metrics' topics where clinicians would benefit from further exposure.*

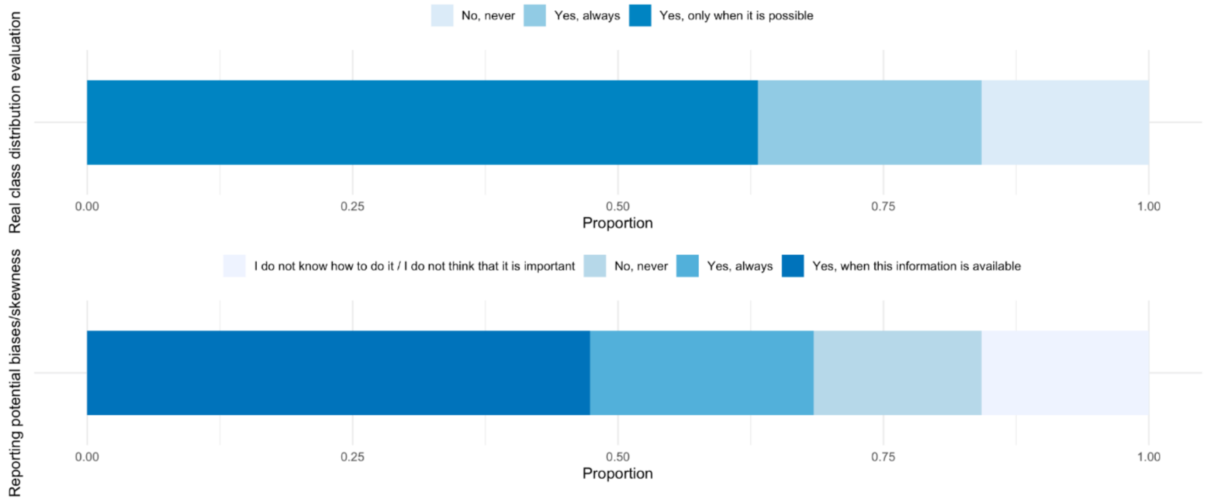

Figure E 6. *Stacked bar plots on the 'Bias and Fairness' topics where clinicians are somehow limited.*

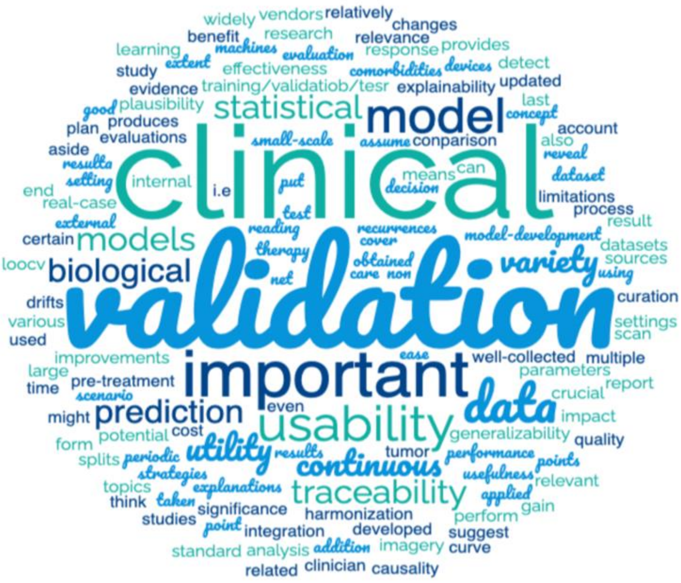

Figure E 7. Word cloud from common terms emerged from open questions on AI validation strategies.

Table E 1 List of themes that emerged from the data analysis and concepts per theme.

Emerging Themes	Concepts
Utility	Usability - Clinical utility - Clinical net benefit<br>Clinical end points - Biological plausibility<br>Causality - ML models for prediction<br>Prediction for decision support - Measures to reduce attention theft and-improve tailored decisions
Capability	Internal validation - Clinical evaluation<br>Data quality - Periodic evaluation<br>Training -Test result comparison<br>Measures to address concept drift
Adoption	Ease of integration into clinical setting - Cost effectiveness
Trustworthiness	Explainability - Traceability

Table E 2 List of themes that emerged from the data analysis and concepts per theme. In the verbatim quotes, 'P' stands for participant.

**Emerging Themes**	Verbatims
Utility	*"Causality, biological plausibility, usefulness and usability, cost effectiveness and ease of integration into clinical settings" (P-18)*<br>*"Potential clinical utility of the results" (P-21)*<br>*"Clinical net benefit is important by means of decision curve analysis" (P-23)*<br>*"Biological models to be put into ML models" (P-17)*<br>*"Pretreatment prediction of the response to therapy of the tumor and the prediction of recurrences" (P-7)*
Capability	*''Internal validation but using well-collected datasets obtained from multiple sources, setting aside generalizability and, in addition, curation provides a good harmonization of the data'' (P-9)*<br>*''Clinical evaluation of a developed model in a real-case scenario (even in the form of a small-scale clinical study) is crucial to reveal limitations and improvements in the dataset used and the learning and model-development validation process''. (P-19)*<br>*"I am wondering if the provided data quality should be examined and reported to the end user, so that the user does not*

	expect magic out of incomplete data and low-quality images. So not only model, but the data validation step is relevant, taking as a benchmark the quality of data used in the model development''. (P-26) *''It is important to perform periodic evaluations to detect data or concept drifts''(P-13)*
Adoption	*"Causality, biological plausibility, usefulness and usability, cost effectiveness and ease of integration into clinical settings" (P-18)*
Trustworthiness	*''Usability, explainability and traceability, the last point is very important, i.e., it is important to perform periodic evaluations to detect data or concept drifts''(P-13)*
